# Diagnostic Accuracy of Panoramic Radiographic Signs for Predicting Inferior Alveolar Nerve Injury Following Mandibular Third Molar Surgery: A Systematic Review and Meta-Analysis

**DOI:** 10.3390/jcm15218315

**Published:** 2026-10-26

**Authors:** Ahmad Othman, Albraa Alolayan

**Affiliations:** Department of Oral and Maxillofacial Diagnostic Sciences, Taibah University, Madina 42353, Saudi Arabia; aalolayan@taibahu.edu.sa

**Keywords:** cone-beam computed tomography, inferior alveolar nerve injuries, mandibular third molar, panoramic radiography, sensitivity and specificity, tooth extraction

## Abstract

**Objectives**: To estimate how accurately individual panoramic radiographic signs predict postoperative inferior alveolar nerve (IAN) injury after mandibular third molar surgery, treating postoperative neurosensory injury, intraoperative nerve exposure and CBCT-defined canal contact as three separate target conditions rather than one. **Methods**: MEDLINE, Embase, Scopus, the Cochrane Library and Google Scholar were searched to 24 June 2025. Risk of bias was assessed with QUADAS-2 and, for randomised trials, RoB 2. Sensitivity and specificity were pooled by sign using DerSimonian–Laird random-effects models and re-examined with a bivariate random-effects model. **Results**: Thirty-seven studies were included; ten contributed two × two data, nine against postoperative neurosensory injury and one against intraoperative exposure. Diversion of the canal discriminated best (specificity 0.95, 95% CI 0.90–0.98; diagnostic odds ratio 8.18, 95% CI 3.31–20.20) but was insensitive (0.27). Only darkening of the root and diversion of the canal withstood removal of any single study. Negative predictive values of 94.2–96.7% added just 0.2–2.5 percentage points beyond pre-test probability, whereas diversion of the canal raised estimated risk from about 5% to 25%. Heterogeneity was substantial (I^2^ 91–98% for specificity), no contributing study was at low risk of bias in all four QUADAS-2 domains, and the reference standard was at high risk in seven of ten. **Conclusions**: Panoramic signs serve risk stratification and are informative when present rather than when absent. The pooled estimates are provisional and should not be transferred to individual practice without allowance for heterogeneity and bias.

## 1. Introduction

Mandibular third molar (MM3) removal is among the most frequently performed procedures in oral and maxillofacial surgery, and impaction is common, with a reported prevalence ranging from 30% to 68% influenced by the angle of eruption and by insufficient space in the arch [1,2]. Removal may be indicated for pericoronitis, caries, periodontal pathology or cyst formation, or as part of orthodontic treatment [3,4]. Injury to the inferior alveolar nerve (IAN) is its most consequential complication. The nerve lies within the mandibular canal and is at greatest risk when the roots of the third molar approach the canal [5]. Temporary sensory disturbance is reported in between 0.26% and 8.4% of cases and permanent deficit in approximately 0.1% to 0.9% [6,7]. Sensory disturbance includes paraesthesia, numbness, dysaesthesia and, in some cases, neuropathic pain of the lower lip, chin or gingiva [8,9]. Most disturbances resolve, but a minority persist and affect oral function and quality of life [10]. IAN injury is also a recurrent source of medicolegal complaint, usually turning on the adequacy of preoperative assessment and consent [11]. Preoperative risk assessment therefore matters both for surgical planning and for what the patient is told beforehand.

Panoramic radiography, also known as orthopantomography (OPG), is the most commonly used initial imaging modality because of its wide availability, relatively low radiation exposure and ability to visualise the mandibular arch in a single examination [2,3]. However, its two-dimensional nature limits assessment of the buccolingual relationship between the MM3 and the inferior alveolar canal (IAC) [5,12]. Several panoramic radiographic signs have nevertheless been associated with an increased risk of postoperative IAN injury, particularly darkening of the root, interruption of the cortical (white) line of the canal and diversion of the canal [10,13,14]. Whether these signs are accurate enough to guide a decision about an individual patient is, however, a different question from whether they are statistically associated with injury across a population, and it is the first question that this review addresses.

Cone-beam computed tomography (CBCT) provides three-dimensional information about the buccolingual relationship between the tooth and the IAC and the integrity of the canal cortex, and may influence surgical planning in selected high-risk cases [15,16]. However, CBCT involves additional radiation exposure and cost and may not be universally available. Importantly, greater anatomical detail does not necessarily translate into a lower rate of postoperative IAN injury, and randomised trials have not consistently demonstrated that routine CBCT reduces postoperative nerve injury compared with panoramic imaging alone [16,17,18,19,20]. The clinically relevant question is therefore not simply whether CBCT provides more anatomical information, but which patients are most likely to benefit from additional three-dimensional imaging.

Several previous syntheses have addressed panoramic prediction of IAN injury. Atieh (2010) and Liu et al. (2015) pooled accuracy across studies without separating the outcome being predicted [3,6]. Su et al. (2017) found darkening of the root, diversion of the canal and interruption of the white line to be associated with postoperative injury but judged the predictive value of individual signs limited [2]. Kang et al. (2020) summarised the risk relationships narratively [7]. Three gaps remain. None of these reviews separated postoperative neurosensory injury from intraoperative nerve exposure and from CBCT-defined tooth–canal contact, although a radiographic sign of proximity predicts proximity far better than it predicts harm. None reported predictive values against the pre-test probability, so the apparently reassuring negative predictive values in this field have not been distinguished from the low event rate that produces them. And none appraised the contributing studies with QUADAS-2 at domain level, so the weight that can be placed on the pooled figures has not been established. The present review addresses those three gaps.

The primary aim of this systematic review and meta-analysis is to estimate the diagnostic accuracy of individual panoramic radiographic signs for predicting postoperative IAN injury after MM3 surgery. Secondary aims are to describe separately the evidence relating panoramic signs to other reference standards, including intraoperative nerve exposure and CBCT-defined canal contact, and to summarise evidence on clinical utility, including whether additional imaging alters referral, surgical approach or the decision to perform coronectomy. Evidence comparing panoramic radiography with CBCT for postoperative nerve injury is also summarised separately. Diagnostic accuracy, anatomical association and clinical utility are treated as distinct questions throughout and are not pooled together.

## 2. Materials and Methods

### 2.1. Protocol and Registration

Three amendments to the registered protocol are declared:

- The registered search end date was extended to 24 June 2025 to capture studies published after registration.
- Risk of bias was to have been assessed with the Newcastle–Ottawa Scale for observational studies. QUADAS-2 was adopted instead as the primary instrument for every study contributing accuracy data, because the review question is one of diagnostic test accuracy and the Newcastle–Ottawa Scale has no domain corresponding to the reference standard; RoB 2 was applied to the randomised trials contributing clinical-utility outcomes. The change replaces a weaker instrument with the one designed for this question and does not alter which studies were eligible.
- Stratification of the pooled analysis by target condition was not pre-specified. It was added after data extraction, once it became clear that the included studies used materially different reference standards and that combining them would pool three different clinical questions. Stratified results are identified as post hoc, and the unstratified analysis is reported in Supplementary Table S5 so that the effect of the amendment is visible.
- PRISMA 2020 checklist is provided as Supplementary Table S1.

### 2.2. Review Question

The review addressed the following question, framed as PIRT (population, index test, reference standard, target condition): in patients undergoing surgical removal of a mandibular third molar (population), what is the accuracy of individual panoramic radiographic signs (index test) for predicting inferior alveolar nerve injury (target condition), as determined by postoperative neurosensory examination, intraoperative visualisation of the nerve, or three-dimensional imaging (reference standards)?

### 2.3. Target Condition and Reference Standards

Included studies used one of three reference standards, which define three distinct target conditions:

- Postoperative IAN injury—altered sensation in the distribution of the inferior alveolar nerve (paraesthesia, hypoaesthesia, dysaesthesia or anaesthesia of the lower lip, chin or gingiva) documented on postoperative clinical neurosensory examination. Studies were accepted where injury was determined by clinical neurosensory examination of the lower lip, chin or gingiva. The specific tests applied, the assessment interval and whether the assessor was blinded to the panoramic findings are recorded per study in Supplementary Table S8, together with the items that the primary reports do not state. No minimum interval could be imposed as an eligibility criterion, because most contributing studies do not report one; the interval actually used is therefore the earliest documented postoperative assessment in each study.
- Intraoperative IAN exposure—direct visualisation of the neurovascular bundle in the extraction socket at operation, without reference to postoperative sensory status. Exposure was accepted as visualisation of the neurovascular bundle in the socket after delivery of the tooth, as judged and recorded by the operating surgeon. It did not require breach of the sheath, bleeding or manipulation of the bundle, and no contributing study reported those events separately from visualisation.
- Radiographic canal contact—absence of an intervening cortical plate between root and canal on CBCT or medical CT, without reference to either operative findings or postoperative sensory status. Contact was accepted where the primary study reported absence of an intervening cortical plate on axial, coronal or sagittal reformats. No contributing study specified a minimum number of slices on which the finding had to be present, and the number of readers and any consensus procedure were reported inconsistently; both are recorded per study in Supplementary Table S8.

These are not interchangeable. Nerve exposure is not nerve injury: a nerve may be visualised intact and remain functionally normal. Radiographic contact is a still weaker surrogate, describing anatomical proximity rather than any clinical event. Because the diagnostic meaning of a panoramic sign depends on which of these it is being tested against, the quantitative synthesis was stratified by target condition and estimates from different reference standards were not combined. The target condition applying to each included study is stated in Table 1 and, for studies contributing two × two data, in Table 2 and Table 3.

Because these operational definitions are reported to differing depths, Supplementary Table S8 sets out for every study contributing two × two data the reference-standard method as reported, the assessment interval, the number of readers and whether interpretation was blinded, and marks unreported items as such rather than inferring them. A reader can therefore judge whether the pooled estimates rest on definitions close enough to those used in their own practice to be worth applying there.

### 2.4. Information Sources and Search

Four bibliographic databases were searched: MEDLINE (via PubMed), Embase, Scopus and the Cochrane Library (CENTRAL). Google Scholar was searched in addition, to capture reports not indexed in those databases. The search covered 1 January 1990 to 24 June 2025, which was the date of the final search in every source. The reference lists of all included studies and of relevant previous systematic reviews were screened by hand. Records retrieved from each source are reported separately in Supplementary Table S4.

**Figure 1:**
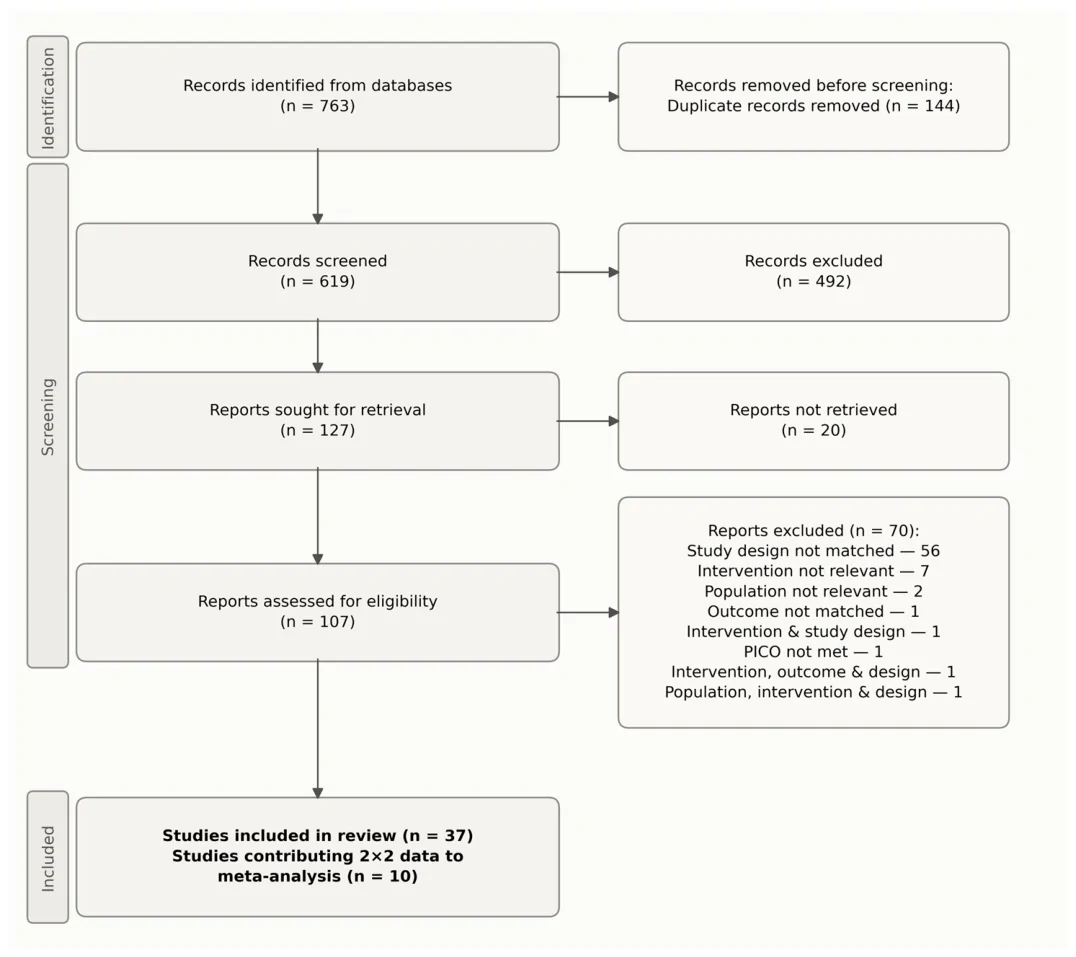
PRISMA 2020 flow diagram.

Retrieval was not restricted by language; the English-language restriction was applied at eligibility assessment, and the number of reports excluded on that ground is given in Figure 1 and Supplementary Table S3. Grey literature was not searched systematically, although the Google Scholar search retrieved 112 records and captured part of it. Conference abstracts, theses and dissertations were excluded by protocol, because they do not report enough methodological detail for QUADAS-2 assessment. Two consequences follow, and they pull in opposite directions. Studies reporting no association between a panoramic sign and nerve injury are less likely to reach an indexed English-language journal, so the pooled diagnostic odds ratios may be biased away from the null; that bias runs against this review’s own conclusion that most signs are of unproven value, which is therefore conservative with respect to it. Evidence from non-English-speaking regions is at the same time under-represented, which limits how widely the estimates apply rather than whether they are internally valid. Neither effect can be quantified from the present data, and no attempt is made to adjust for either.

The full search string for every source, with the number of records retrieved from each, is reproduced in Supplementary Table S4. Three concept blocks were combined with AND in every source: third molar, panoramic radiography, and inferior alveolar nerve injury or the mandibular canal. No methodological filter and no study-design limit was applied.

### 2.5. Eligibility Criteria

Eligibility criteria are stated once here. Studies were included if they met all of the following:

- Population—patients of any age or sex undergoing surgical removal of an impacted mandibular third molar, with a preoperative panoramic radiograph available.
- Index test—assessment of one or more panoramic radiographic signs of proximity between the third molar and the inferior alveolar canal.
- Reference standard—postoperative neurosensory examination, intraoperative visalisation of the nerve, or CBCT/CT assessment of the canal–root relationship.
- Outcome data—sufficient data to construct a two × two table, or explicitly reported sensitivity, specificity, positive predictive value or negative predictive value.
- Design—randomised controlled trial, prospective or retrospective cohort study, cross-sectional study, or diagnostic accuracy study, published in a peer-reviewed journal.

Studies were excluded if they were case reports, case series or had a sample size below 10; were reviews, editorials, letters, conference abstracts or theses; did not report IAN status or its association with panoramic signs; were animal or in vitro studies; or were unavailable in full text or not published in English.

### 2.6. Index Test: Definition of the Panoramic Signs

Seven panoramic radiographic signs were assessed. Six are the criteria described by Rood and Shehab [21] darkening of the root, interruption of the white line of the canal, diversion of the canal, narrowing of the canal, narrowing of the root, and deflection of the root together with a dark and bifid root apex, which several later studies report alongside them. These seven were selected a priori because they are the only signs reported with extractable two × two data in more than two included studies; signs reported by one or two studies only (for example juxta-apical radiolucency) are described narratively and were not pooled. Each sign was recorded as present or absent on the preoperative panoramic radiograph as defined by the authors of the primary study.

Definitions of the signs were obtained from the primary studies and were not harmonised, because the original radiographs were not available for re-reading. This has a predictable effect on the pooled estimates. Where a sign is defined liberally, more radiographs are scored positive, raising sensitivity and lowering specificity; where it is defined strictly, the reverse. Across studies, this produces variation in the implicit threshold at which a sign is called present rather than in the underlying discriminative ability of the sign, and it appears as heterogeneity concentrated in specificity rather than in sensitivity. That is the pattern observed here (Section 3.4). Two consequences follow for interpretation. The pooled sensitivity and specificity of a sign describe an average threshold that no individual reader necessarily applies, whereas the diagnostic odds ratio and the summary receiver operating characteristic curve depend less on threshold and are the more transferable summaries. And because inter-observer agreement and an explicit operational definition were reported too rarely to serve as covariates, the size of this effect could not be estimated by meta-regression and is stated as a direction rather than a magnitude.

### 2.7. Study Selection and Data Extraction

Study selection was carried out in two stages. Two independent reviewers screened titles and abstracts to identify potentially relevant articles. The full texts of shortlisted articles were then screened against the eligibility criteria. Discrepancies were settled by consensus or, if necessary, by a third reviewer. Data were extracted independently by two reviewers using a piloted form. Corresponding authors were contacted where data were missing or unclear.

For every sign in every study, the number of true positives (TP; sign present and target condition present), true negatives (TN; sign absent and target condition absent), false positives (FP; sign present and target condition absent) and false negatives (FN; sign absent and target condition present) was extracted or reconstructed from reported accuracy statistics and sample sizes. The unit of analysis is stated for each study in Leungs 2 and 3 and is either the patient or the tooth, according to how the primary study reported its data. Five of the nine studies in the primary analysis reported by tooth or molar (Rood and Shehab 1990 [21], Gomes et al. 2008 [22], Leung and Cheung 2011 [23], Ghaeminia et al. 2015 [16], Hasegawa et al. 2013 [24]) and four reported by patient (Huang et al. 2015 [25], Gallesio et al. 2010 [26], Harada et al. 2015 [27], Szalma et al. 2010 [10]). Teeth from the same patient are not independent, and none of the five tooth-level studies reports how many patients contributed more than one tooth. A design effect could therefore not be calculated and no variance inflation was applied, with the consequence that the confidence intervals for any estimate drawing on tooth-level studies are narrower than the data strictly support; the point estimates are unaffected. Two steps were taken instead of an adjustment. No patient or tooth contributes more than once to any single pooled estimate, and a sensitivity analysis restricted to the patient-level studies was performed for every sign and is reported in Section 3.9. Every extracted two × two table was checked for internal consistency, in that the four cells were required to sum to the reported denominator and the number of diseased units was required to be the same across all signs reported by that study. Rows failing these checks are identified in Table 2 and their handling is described in Section 2.9 and Supplementary Table S5.

### 2.8. Risk of Bias

Risk of bias was assessed with the instrument matched to each study’s design and to its role in the review, so that one instrument governs each class of study. QUADAS-2 [28] was applied to every study contributing diagnostic accuracy data, because the review question is one of test accuracy. Its four domains, namely patient selection, index test, reference standard and flow and timing, were rated low, high or unclear, with applicability rated for the first three. Two signalling questions were tailored to this review: whether panoramic signs were interpreted without knowledge of the reference standard result, and whether nerve status was assessed without knowledge of the panoramic findings. Domain-level judgements are reported in Table 8 and the basis for each judgement in Supplementary Table S7.

Randomised trials comparing CBCT with panoramic radiography address clinical utility rather than accuracy, so they were appraised with RoB 2 [29], are reported separately and contribute no data to any pooled accuracy estimate. The Newcastle–Ottawa Scale was retained only as a descriptive appraisal of the observational cohorts that contributed no two × two data, and no Newcastle–Ottawa score supports any accuracy conclusion in this review.

### 2.9. Statistical Analysis

Analyses were performed in Python 3.11 (NumPy 2.4, SciPy 1.17). For each radiographic sign, study-level sensitivity and specificity were logit-transformed and pooled using DerSimonian–Laird random-effects models [30], with between-study variance (τ^2^) estimated by the method of moments. A continuity correction of 0.5 was added to all four cells of any two × two table containing a zero. Pooled estimates were back-transformed to the proportion scale and are reported with 95% confidence intervals. Heterogeneity is reported as I^2^ for both sensitivity and specificity; prediction intervals were computed where at least three studies contributed and τ^2^ exceeded zero.

DerSimonian–Laird was chosen as the primary method for two reasons. It is estimable for every sign in this dataset, including the four reported by only three to five studies, and it does not require the between-study correlation of sensitivity and specificity to be identified from the data. Its known limitation is the one the reviewers of this manuscript raise: with few studies, τ^2^ is imprecisely estimated and the resulting confidence intervals are too narrow. Three measures were taken in response. Prediction intervals are reported alongside the confidence intervals (Table 5); every pooled estimate was re-examined with a bivariate random-effects model, described below; and no pooled estimate is interpreted as a transferable property of the test.

Diagnostic odds ratios were pooled on the log scale using the same random-effects model. Positive and negative likelihood ratios were derived from the pooled sensitivity and specificity. Summary receiver operating characteristic (SROC) curves were fitted using the Moses–Littenberg method [31], regressing D = logit(sensitivity) + logit(specificity) on S = logit(sensitivity) − logit(specificity), weighted by the inverse variance of D. The slope was tested against zero; where it did not differ significantly from zero, the symmetric model was fitted. Because the Moses–Littenberg model is unstable when few studies contribute over a narrow threshold range, the area under the curve was reported only for signs with at least seven contributing studies and was considered not estimable otherwise.

Each sign was additionally analysed with a bivariate random-effects model, fitted by maximum likelihood to the logit-transformed sensitivity and specificity jointly and estimating the two between-study standard deviations together with their correlation [32]. The fitted parameters were reparameterised to the hierarchical summary receiver operating characteristic scale to obtain an area under the curve [33]. This model was treated as confirmatory rather than primary. It was fitted where at least four studies contributed, and a solution was accepted only where the correlation was estimated inside its admissible range and the information matrix could be inverted; where either condition failed, the estimate is reported as not estimable rather than as a number. Results are in Table 5.

Pooled prevalence, positive predictive value (PPV) and negative predictive value {NPV} were calculated from the summed two × two cells across contributing studies, with Wilson score confidence intervals [34]. Because predictive values depend on prevalence, each is also reported as the absolute change in percentage points from the corresponding pre-test probability: for PPV, the change from the prevalence of the target condition; for NPV, the change from the pre-test probability of no injury (100 − prevalence). Interpretation therefore considered the incremental change in probability rather than the absolute predictive value alone. Predictive values were therefore not derived from the pooled sensitivity and specificity at an assumed prevalence. They are the observed proportions in the pooled two × two data, and the prevalence to which each refers is the pooled prevalence reported beside it in Table 6. Because that prevalence is a property of the contributing studies rather than of general practice, post-test probabilities were also computed from the pooled likelihood ratios across a range of baseline risks and are reported in Table 7.

All analyses were stratified by target condition. Estimates derived from studies using postoperative neurosensory injury as the reference standard constituted the primary analysis. Studies using intraoperative nerve exposure were reported separately and were not combined with the primary analysis. No study contributing two × two data used CBCT-defined canal contact as the reference standard; therefore, no pooled estimate was produced for that target condition.

Two extracted rows failed the internal consistency checks described in Section 2.7. One was a transcription duplicate and was removed, so narrowing of the canal is pooled from four studies rather than five; the other could not be resolved with certainty, and all four arithmetically admissible reconstructions were carried through the analysis (Section 3.9). The rows concerned, the reasoning behind each decision and the full reconstruction set are documented in Supplementary Tables S5 and S10.

Publication bias was not formally assessed. Funnel plot asymmetry tests have limited reliability in diagnostic accuracy meta-analysis, particularly when few studies contribute to each pooled estimate. No radiographic sign was reported by ten or more studies; therefore, the previously proposed use of Egger’s test was not pursued.

Because no study contributing two × two data was at low risk of bias across all four QUADAS-2 domains, a sensitivity analysis restricted to studies at overall low risk of bias was not feasible. Four sensitivity analyses were performed instead: leave-one-out analysis for every sign, reporting the range of the diagnostic odds ratio and of both likelihood ratios across iterations; an analysis restricted to the studies using the patient as the unit of analysis; an analysis excluding Szalma et al. (2010) [10], the one contributing study with a case–control sampling design; and an analysis of the four arithmetically admissible reconstructions of the internally inconsistent Leung and Cheung (2011) [23] row. Meta-regression was not performed. With three to eight studies per sign, there is no prospect of reliable estimation, and with covariates reported as coarsely as they are here a fitted model would mislead rather than explain.

## 3. Results

### 3.1. Study Selection

A total of 763 records were identified from the database search, with removal of 144 duplicates before screening. In total, 619 records were screened by title and abstract, of which 492 were excluded as not meeting the eligibility criteria. Full text was sought for 127 reports; 20 could not be retrieved, leaving 107 reports assessed for eligibility. Of these, 70 were excluded, with reasons recorded in Supplementary Table S3. Thirty-seven studies met all inclusion criteria and were included in the review. Ten of these reported data sufficient to construct a two × two table and contributed to the meta-analysis; the remaining 27 contributed to the narrative synthesis only. The PRISMA 2020 flow diagram is shown in Figure 1.

All 27 studies that did not contribute to the meta-analysis were excluded from it for the same single reason: none reported, or permitted reconstruction of, a two × two table for an individual panoramic sign against one of the three reference standards defined in Section 2.3. Most reported only an odds ratio, a correlation coefficient or a *p* value for the association, and several reported accuracies for a composite of signs rather than for any sign separately. They contribute to the narrative synthesis and are listed with the other included studies in Table 1.

### 3.2. Study Characteristics

The 37 included studies were published between 1990 and 2024, and 26 appeared after 2010. Sample sizes ranged from 31 to 4917 extraction sites. Every study used panoramic radiography as the index test. Study designs were retrospective cohort (16), prospective cohort (10), randomised controlled trial (4), diagnostic accuracy or comparative study (4), cross-sectional (2) and retrospective case–control (1).

Studies differed in the reference standard used, and this distinction organises the synthesis that follows. Twenty studies used postoperative neurosensory examination, seven used intraoperative visualisation of the nerve and ten used CBCT or medical CT assessment of the canal–root relationship; several reported more than one, in which case the standard against which two × two data were extracted is the one recorded in Table 2. Of the ten studies contributing pooled data, nine used postoperative neurosensory injury and one used intraoperative nerve exposure. No study contributing pooled data used CBCT-defined canal contact. The pooled estimates in Section 3.4 therefore rest on a clinically homogeneous target condition, and the CBCT-correlation literature is summarised narratively in Section 3.6 without being combined with them.

Studies were conducted in Asia, Europe, North America, South America and Australia. Follow-up, where reported, ranged from immediate postoperative review to several months. Study characteristics are given in Table 1, which reports design, population, reference standard and target condition for every included study; intervention, comparator, outcomes measured, key findings and limitations are given in Supplementary Table S6. Geographic distribution is reported here only and is not repeated later in the Results.

**Table 1:** Design, population and reference standard of the 37 included studies. The target condition column states which of the three reference standards defined in Section 2.3 each study used. Intervention, comparator, outcomes measured, key findings and limitations for all 37 studies are provided in Supplementary Table S6.

#	Study (Year)	Design	Population	Reference Standard	Target Condition
1	Tantanapornkul (2007) [35]	Prospective cohort	120 patients (142 teeth)	Intraoperative nerve exposure	Intraoperative IAN exposure
2	Khemla and Wongchuensoontorn (2022) [36]	Retrospective cohort	106 patients (154 teeth)	CBCT/CT canal contact	Radiographic canal contact
3	Elkhateeb and Awad (2018) [12]	Retrospective cohort	135 patients (210 molars)	CBCT/CT canal contact	Radiographic canal contact
4	Lee (2015) [37]	Retrospective cohort	3555 patients (4917 extractions)	Postoperative neurosensory examination	Postoperative IAN injury
5	Guerrero (2014) [17]	Randomised controlled trial	256 patients	Postoperative neurosensory examination	Postoperative IAN injury
6	Harada (2015) [27]	Retrospective cohort	280 patients	Postoperative neurosensory examination	Postoperative IAN injury
7	Nakamori (2008) [38]	Retrospective cohort	443 patients (695 teeth)	CBCT/CT canal contact	Radiographic canal contact
8	Ghaeminia (2015) [16]	Randomised controlled trial	268 patients (320 molars)	Postoperative neurosensory examination	Postoperative IAN injury
9	Jhamb (2009) [39]	Diagnostic accuracy/comparative	31 molars	Intraoperative nerve exposure	Intraoperative IAN exposure
10	Luo (2018) [40]	Cross-sectional	479 patients	Postoperative neurosensory examination	Postoperative IAN injury
11	Leung & Cheung (2011) [23]	Prospective cohort	118 patients (178 teeth)	Postoperative neurosensory examination	Postoperative IAN injury
12	Hasani (2017) [41]	Prospective cohort	59 extraction sites	Intraoperative nerve exposure	Intraoperative IAN exposure
13	Vranckx (2022) [42]	Prospective cohort	201 patients (357 molars)	Postoperative neurosensory examination	Postoperative IAN injury
14	Korkmaz (2017) [43]	Prospective cohort	122 patients (139 molars)	Postoperative neurosensory examination	Postoperative IAN injury
15	Savani (2018) [44]	Prospective cohort	60 patients (120 molars)	CBCT/CT canal contact	Radiographic canal contact
16	Yuce (2021) [45]	Diagnostic accuracy/comparative	150 patients	CBCT/CT canal contact	Radiographic canal contact
17	Kubota (2023) [46]	Diagnostic accuracy/comparative	1573 patients	Postoperative neurosensory examination	Postoperative IAN injury
18	Selvi (2013) [47]	Retrospective cohort	149 patients (235 molars)	Postoperative neurosensory examination	Postoperative IAN injury
19	Jerjes (2006) [48]	Retrospective cohort	1934 patients	Postoperative neurosensory examination	Postoperative IAN injury
20	Winstanley (2018) [13]	Retrospective cohort	55 patients (100 molars)	CBCT/CT canal contact	Radiographic canal contact
21	Guerrero (2012) [49]	Randomised controlled trial	79 patients (86 molars)	Postoperative neurosensory examination	Postoperative IAN injury
22	Petersen (2016) [18]	Randomised controlled trial	230 patients	Postoperative neurosensory examination	Postoperative IAN injury
23	Sedaghatfar (2005) [50]	Retrospective cohort	230 patients (423 molars)	Intraoperative nerve exposure	Intraoperative IAN exposure
24	Mansour (2012) [51]	Retrospective cohort	100 patients	Intraoperative nerve exposure	Intraoperative IAN exposure
25	Ghaeminia (2009) [52]	Prospective cohort	40 patients (53 molars)	CBCT/CT canal contact	Radiographic canal contact
26	Susarla and Dodson (2007) [53]	Retrospective cohort	23 patients (46 molars)	Intraoperative nerve exposure	Intraoperative IAN exposure
27	Bandiwadekar (2024) [54]	Retrospective cohort	100 patients	Intraoperative nerve exposure	Intraoperative IAN exposure
28	Rood and Shehab (1990) [21]	Retrospective cohort	1560 molars	Postoperative neurosensory examination	Postoperative IAN injury
29	Kapila (2014) [55]	Retrospective cohort	42 patients	CBCT/CT canal contact	Radiographic canal contact
30	Azahar (2018) [56]	Prospective cohort	32 patients (35 molars)	CBCT/CT canal contact	Radiographic canal contact
31	Picoli (2023) [57]	Prospective cohort	25 patients (50 molars)	Postoperative neurosensory examination	Postoperative IAN injury
32	Hasegawa (2013) [24]	Retrospective cohort	295 patients (440 molars)	Postoperative neurosensory examination	Postoperative IAN injury
33	Gomes (2008) [22]	Diagnostic accuracy/comparative	153 patients (260 molars)	Postoperative neurosensory examination	Postoperative IAN injury
34	Gallesio (2010) [26]	Prospective cohort	134 patients	Postoperative neurosensory examination	Postoperative IAN injury
35	Ruiz-Roca (2024) [58]	Cross-sectional	305 patients (332 molars)	CBCT/CT canal contact	Radiographic canal contact
36	Huang (2015) [25]	Retrospective cohort	120 patients	Postoperative neurosensory examination	Postoperative IAN injury
37	Szalma (2010) [10]	Retrospective case–control	309 patients	Postoperative neurosensory examination	Postoperative IAN injury

### 3.3. Two × Two Data Contributing to the Meta-Analysis

Ten studies reported extractable two × two data for one or more of the seven panoramic signs (Table 2). Each row states the unit of analysis and the target condition, so that the reader can see which studies contribute to which pooled estimate and on what basis. No patient or tooth contributes more than once to any single pooled estimate. The two rows that failed the internal consistency checks of Section 2.7 are identified in Table 2; their handling and the alternative reconstructions considered are set out in Section 3.9 and Supplementary Tables S5 and S10.

**Table 2:** Two × two data extracted for each panoramic radiographic sign, by study, with unit of analysis and target condition. TP, true positive; TN, true negative; FP, false positive; FN, false negative.

Study (Year) [Ref]	Country	Unit of Analysis	Target Condition	Radiographic Sign	TP	TN	FP	FN
Huang 2015 [25]	China	Patient	Postoperative IAN injury	Darkening of the root	7	72	35	6
				Interruption of the white line	12	49	58	1
				Diversion of the canal	10	90	17	3
				Dark and bifid root apex	3	85	22	10
				Deflection of the root	4	83	24	9
				Narrowing of the canal	9	70	37	4
				Narrowing of the root	3	84	23	10
Gomes 2008 [22]	Brazil	Molar	Postoperative IAN injury	Darkening of the root	3	201	50	6
				Interruption of the white line	2	236	15	7
				Diversion of the canal	1	247	4	8
Rood 1990 [21]	UK	Molar	Postoperative IAN injury	Darkening of the root	8	701	38	13
				Interruption of the white line	5	709	30	16
				Diversion of the canal	6	727	12	15
				Narrowing of the root	0	737	2	21
Leung 2011 [23]	China	Tooth	Postoperative IAN injury	Darkening of the root	6	125	44	3
				Narrowing of the root	1	165	4	8
				Interruption of the white line	9	158	11	0
				Diversion of the canal	1	147	22	8
				Narrowing of the canal	0	160	9	9
Ghaeminia 2015 [16]	Netherlands	Molar	Postoperative IAN injury	Darkening of the root	15	214	74	5
				Interruption of the white line	5	215	73	15
Gallesio 2010 [26]	Italy	Patient	Postoperative IAN injury	Diversion of the canal	0	122	8	4
				Narrowing of the canal	0	124	6	4
				Deflection of the root	0	121	9	4
				Narrowing of the root	0	124	6	4
				Dark and bifid root apex	0	121	9	4
				Interruption of the white line	1	91	39	3
				Darkening of the root	3	98	32	1
Sedaghatfar 2005 [50]	USA	Molar	Intraoperative IAN exposure	Interruption of the white line	18	265	134	6
				Diversion of the canal	10	356	43	14
				Deflection of the root	4	344	55	20
				Narrowing of the root	12	361	38	12
Hasegawa 2013 [24]	Japan	Molar	Postoperative IAN injury	Interruption of the white line	9	399	13	19
				Diversion of the canal	11	395	17	17
Harada 2015 [27]	Japan	Patient	Postoperative IAN injury	Darkening of the root	3	174	91	12
				Interruption of the white line	3	124	141	12
				Diversion of the canal	0	259	6	15
				Dark and bifid root apex	0	262	3	15
				Deflection of the root	1	263	2	14
				Narrowing of the canal	0	255	10	15
				Narrowing of the root	0	263	2	15
Szalma 2010 [10]	Hungary	Patient	Postoperative IAN injury	Darkening of the root	32	178	84	15

The ten studies contributing two × two data differ from one another in ways that bear directly on what the pooled estimates mean, and Table 3 sets those differences out before the estimates are presented. They span from 1990 to 2015 and eight countries on four continents. Six analysed teeth or molars and four analysed patients; one of the four, Szalma et al. (2010) [10], sampled cases and controls rather than a clinical series, so the event rate in that study is set by its design. Nine used postoperative neurosensory examination as the reference standard, and one used intraoperative exposure. The method of neurosensory assessment is described in only one study, and the interval at which it was performed in only two—one week in Harada et al. (2015) [27] and up to six months in Hasegawa et al. (2013) [24]. Only Hasegawa et al. report that radiographic interpretation was blinded to nerve status. The number of signs reported per study ranges from one to seven, so no two pooled estimates rest on quite the same subset of the ten studies, and the k column in Table 4 should be read with Table 3 beside it.

**Table 3:** Clinical and methodological diversity among the ten studies contributing two × two data. Blinding of radiographic interpretation to nerve status is reported by one study only (Hasegawa et al. 2013 [24]); the remainder do not state it. Full operational detail, including the number of readers, is given in Supplementary Table S8. IAN, inferior alveolar nerve.

Study (Year) [Ref]	Design	Country	Unit of Analysis	Target Condition	Reference Standard as Reported	Assessment Interval	Signs with 2 × 2 Data
Huang 2015 [25]	Retrospective cohort	China	Patient	Postoperative IAN injury	Clinical neurosensory examination; method not detailed	Not reported	7
Gomes 2008 [22]	Diagnostic accuracy	Brazil	Molar	Postoperative IAN injury	Clinical neurosensory examination; method not detailed	Not reported	3
Rood 1990 [21]	Retrospective cohort	UK	Molar	Postoperative IAN injury	Postoperative sensory assessment; not standardised or described	Not reported	4
Leung 2011 [23]	Prospective cohort	China	Tooth	Postoperative IAN injury	Postoperative neurosensory deficit; method not described	Not reported	5
Ghaeminia 2015 [16]	Randomised controlled trial	Netherlands	Molar	Postoperative IAN injury	Altered sensation self-reported by unblinded participants	Not reported	3
Gallesio 2010 [26]	Prospective cohort	Italy	Patient	Postoperative IAN injury	Clinical neurosensory examination; method not detailed	Not reported	7
Sedaghatfar 2005 [50]	Retrospective cohort	USA	Molar	Intraoperative IAN exposure	Intraoperative visualisation recorded by the operating surgeon	At operation	4
Hasegawa 2013 [24]	Retrospective cohort	Japan	Molar	Postoperative IAN injury	Pin-prick and light-touch testing with neurological examination	Up to 6 months	2
Harada 2015 [27]	Retrospective cohort	Japan	Patient	Postoperative IAN injury	Paraesthesia as reported; no standardised protocol described	1 week	7
Szalma 2010 [10]	Retrospective case–control	Hungary	Patient	Postoperative IAN injury	Postoperative paraesthesia; ascertainment not described	Not reported	1

**Table 4:** Pooled diagnostic accuracy of panoramic radiographic signs for postoperative inferior alveolar nerve injury. k, number of contributing studies; N, total units analysed; LR+, positive likelihood ratio; LR−, negative likelihood ratio; DOR, diagnostic odds ratio; AUC, area under the summary receiver operating characteristic curve; NE, not estimable (fewer than seven contributing studies). Estimates are DerSimonian–Laird random-effects pooled values on the logit scale.

Radiographic Sign	k	N	Sensitivity (95% CI)	I^2^, %	Specificity (95% CI)	I^2^, %	LR+	LR−	DOR (95% CI)	AUC
Darkening of the root	8	2349	0.54 (0.38–0.68)	59	0.77 (0.67–0.85)	95	2.32	0.60	3.92 (1.97–7.80)	0.73
Interruption of the white line	8	2480	0.37 (0.21–0.56)	63	0.84 (0.67–0.94)	98	2.35	0.75	3.96 (1.09–14.39)	0.70
Diversion of the canal	7	2172	0.27 (0.12–0.49)	65	0.95 (0.90–0.98)	91	5.66	0.77	8.18 (3.31–20.20)	0.84
Narrowing of the canal	4	712	0.15 (0.02–0.67)	77	0.92 (0.72–0.98)	96	1.80	0.93	2.67 (0.98–7.28)	NE
Narrowing of the root	5	1472	0.12 (0.05–0.26)	0	0.97 (0.88–0.99)	94	4.60	0.90	2.10 (0.79–5.60)	NE
Deflection of the root	3	534	0.18 (0.06–0.42)	25	0.94 (0.76–0.99)	93	2.97	0.88	2.12 (0.74–6.07)	NE
Dark and bifid root apex	3	534	0.15 (0.05–0.36)	6	0.94 (0.76–0.98)	93	2.34	0.91	1.33 (0.42–4.21)	NE
*Intraoperative nerve exposure—reported separately (Sedaghatfar et al. 2005 [50]; not pooled with the above)*										
Interruption of the white line	1	423	0.75 (0.54–0.88)	—	0.66 (0.62–0.71)	—	2.23	0.38	5.93 (2.30–15.30)	NE
Diversion of the canal	1	423	0.42 (0.24–0.62)	—	0.89 (0.86–0.92)	—	3.87	0.65	5.91 (2.47–14.13)	NE
Narrowing of the root	1	423	0.50 (0.31–0.69)	—	0.90 (0.87–0.93)	—	5.25	0.55	9.50 (3.99–22.61)	NE
Deflection of the root	1	423	0.17 (0.06–0.37)	—	0.86 (0.82–0.89)	—	1.21	0.97	1.25 (0.41–3.80)	NE

### 3.4. Pooled Diagnostic Accuracy: Postoperative Inferior Alveolar Nerve Injury

**Figure 2:**
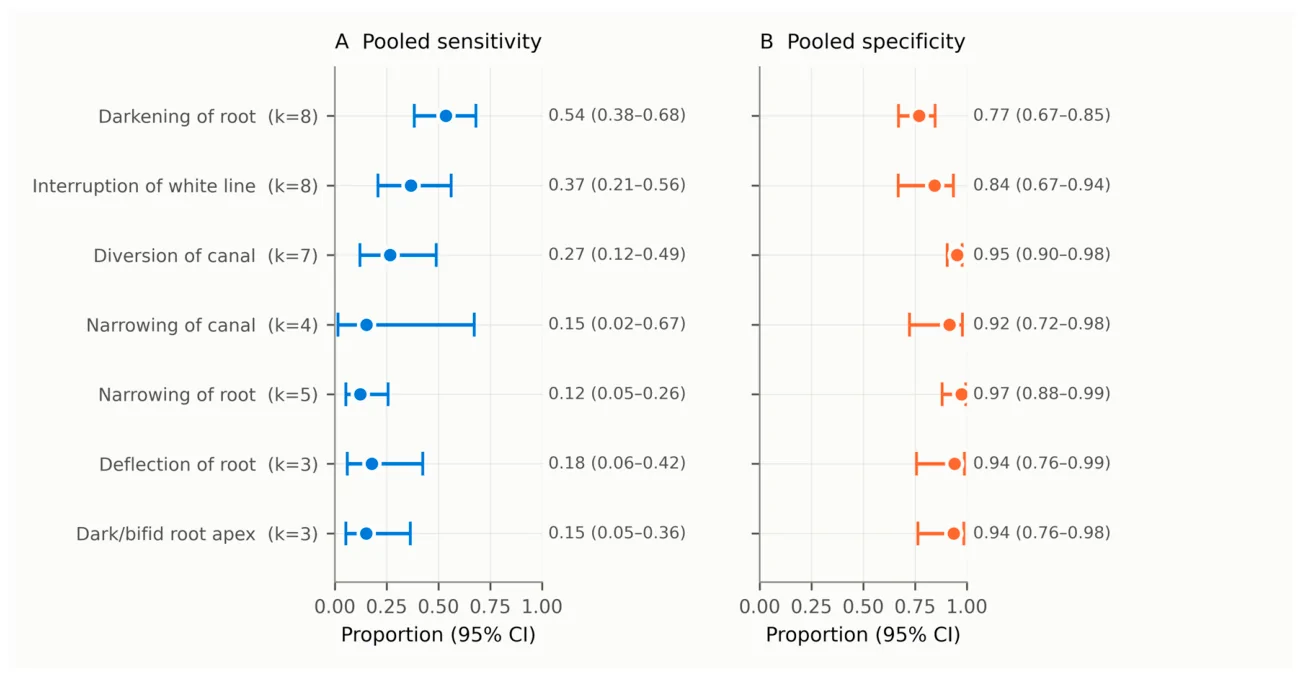
Pooled sensitivity (**A**) and specificity (**B**) with 95% confidence intervals for each panoramic radiographic sign, for the target condition of postoperative inferior alveolar nerve injury. k denotes the number of contributing studies.

The primary analysis pools the nine studies that used postoperative neurosensory examination as the reference standard (Table 4, Figure 2). Darkening of the root was the most sensitive sign, at 0.54 (95% CI 0.38–0.68), pooled from eight studies and 2349 units; it was also the least specific, at 0.77 (95% CI 0.67–0.85). Diversion of the canal was the most specific, at 0.95 (95% CI 0.90–0.98) from seven studies, with sensitivity of only 0.27 (95% CI 0.12–0.49). Interruption of the white line fell between the two, with sensitivity 0.37 (95% CI 0.21–0.56) and specificity 0.84 (95% CI 0.67–0.94).

**Figure 3:**
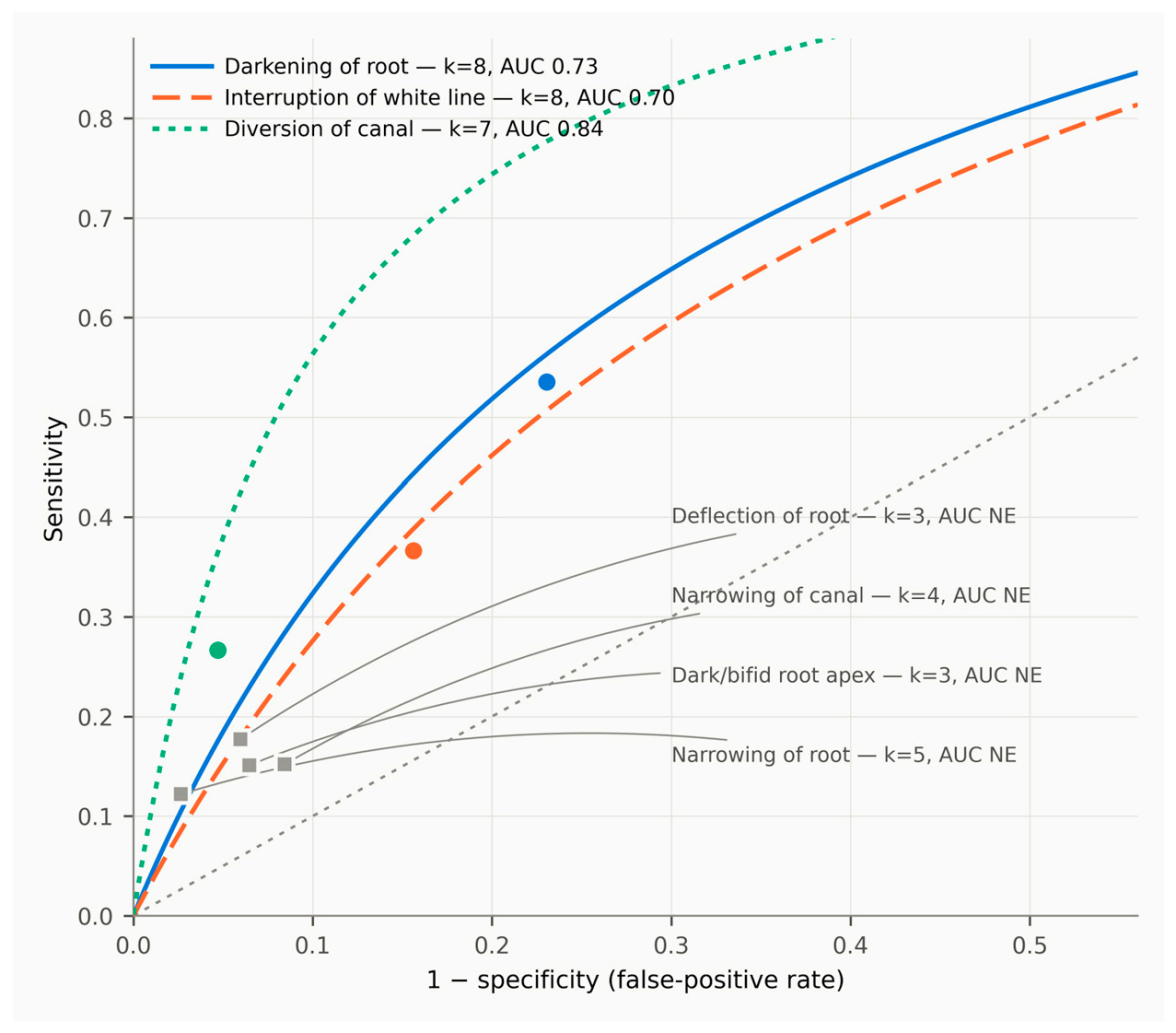
Summary receiver operating characteristic curves for the three panoramic signs with at least seven contributing studies, with summary operating points for all seven signs. The area under the curve is reported as not estimable (NE) for signs with fewer than seven contributing studies, because the Moses–Littenberg model is unstable over a narrow threshold range.

Diversion of the canal had the strongest overall discrimination, with a positive likelihood ratio of 5.66, a diagnostic odds ratio of 8.18 (95% CI 3.31–20.20) and an SROC area under the curve of 0.84. Darkening of the root and interruption of the white line had areas under the curve of 0.73 and 0.70, respectively. For the remaining four signs, the Moses–Littenberg model was not stable enough to report an area under the curve, and the diagnostic odds ratio is the summary statistic SROC results are shown in Figure 3.

Only three of the seven signs had a diagnostic odds ratio whose 95% confidence interval excluded 1: darkening of the root (3.92; 1.97–7.80), interruption of the white line (3.96; 1.09–14.39) and diversion of the canal (8.18; 3.31–20.20). Of these three, only darkening of the root and diversion of the canal retained significance in leave-one-out analysis (Section 3.7). Narrowing of the canal (2.67; 0.98–7.28), narrowing of the root (2.10; 0.79–5.60), deflection of the root (2.12; 0.74–6.07) and a dark and bifid root apex (1.33; 0.42–4.21) were each compatible with no association. These four signs are pooled from three to five studies only and should not be presented as predictors of nerve injury on the present evidence.

Heterogeneity was substantial. I^2^ for specificity ranged from 91% to 98% across the seven signs, and I^2^ for sensitivity from 0% to 77%. This reflects genuine differences in how each sign was defined and read, in surgical technique and operator experience, and in how neurosensory outcome was ascertained and over what interval. The pooled estimates should be read as a summary of a heterogeneous body of evidence rather than as a single transferable accuracy figure, and the confidence intervals in Table 4 do not capture that heterogeneity in full. Sensitivity, specificity and the area under the curve reported here are properties of this collection of studies rather than constants of the test. With I^2^ for specificity between 91% and 98% they should not be carried unchanged into another population, another reader or another definition of the sign, and the prediction intervals in Table 5, which are wide, are a better guide than the confidence intervals to what a new study might observe.

The bivariate random-effects model reproduced the primary estimates closely wherever it could be fitted (Table 5). For the three signs with at least seven contributing studies, darkening of the root, interruption of the white line and diversion of the canal pooled sensitivity and specificity agreed with the DerSimonian–Laird estimates to within 0.04, and the between-study correlation between sensitivity and specificity was negative in every case, from −0.28 to −0.72, which is what would be expected if studies differ mainly in the implicit threshold at which a sign is called present. The hierarchical summary receiver operating characteristic areas under the curve were 0.70 for darkening of the root, 0.81 for diversion of the canal and 0.58 for interruption of the white line. The first two are close to the Moses–Littenberg values in Table 4; for interruption of the white line the Moses–Littenberg estimate of 0.70 is the more optimistic of the two and should not be relied upon. For narrowing of the canal and narrowing of the root, the model converged only with the correlation on its boundary, and for deflection of the root and a dark and bifid root apex, each with three studies, it could not be fitted at all. Two conclusions follow. The findings of this review do not depend on the choice of pooling method, and the four signs for which the bivariate model is not estimable are the same four for which no association was demonstrated; the limitation is in the evidence, not in the model.

**Table 5:** Bivariate random-effects estimates, between-study correlation, hierarchical summary receiver operating characteristic (HSROC) area under the curve, and DerSimonian–Laird 95% prediction intervals, for postoperative inferior alveolar nerve injury. k, number of contributing studies; ρ, estimated between-study correlation of logit sensitivity and logit specificity; PI, prediction interval; NE, not estimable. The bivariate model was fitted where at least four studies contributed and accepted only where ρ was estimated inside its admissible range and the information matrix could be inverted.

Radiographic Sign	k	Bivariate Sensitivity (95% CI)	Bivariate Specificity (95% CI)	ρ (Sensitivity, Specificity)	HSROC AUC	95% PI, Sensitivity	95% PI, Specificity	Model Status
Darkening of the root	8	0.54 (0.40–0.67)	0.77 (0.67–0.84)	−0.42	0.70	0.16–0.87	0.35–0.95	Converged
Interruption of the white line	8	0.36 (0.19–0.57)	0.84 (0.68–0.93)	−0.28	0.58	0.05–0.85	0.12–1.00	Converged
Diversion of the canal	7	0.31 (0.12–0.59)	0.95 (0.91–0.98)	−0.72	0.81	0.02–0.86	0.58–1.00	Converged
Narrowing of the canal	4	NE	NE	−0.99 (boundary)	NE	0.00–1.00	0.01–1.00	Correlation on boundary; not reported
Narrowing of the root	5	NE	NE	−0.99 (boundary)	NE	Not estimable	0.07–1.00	Correlation on boundary; not reported
Deflection of the root	3	NE	NE	NE	NE	0.00–1.00	0.00–1.00	Not fitted (k < 4)
Dark and bifid root apex	3	NE	NE	NE	NE	0.00–1.00	0.00–1.00	Not fitted (k < 4)

### 3.5. Predictive Values and Their Dependence on Prevalence

Pooled predictive values are reported in Table 4. Positive predictive values were low: 25.2% for diversion of the canal (95% CI 18.2–33.9) and between 8.1% and 14.7% for the other six signs. Negative predictive values were uniformly high, between 94.2% and 96.7%.

**Figure 4:**
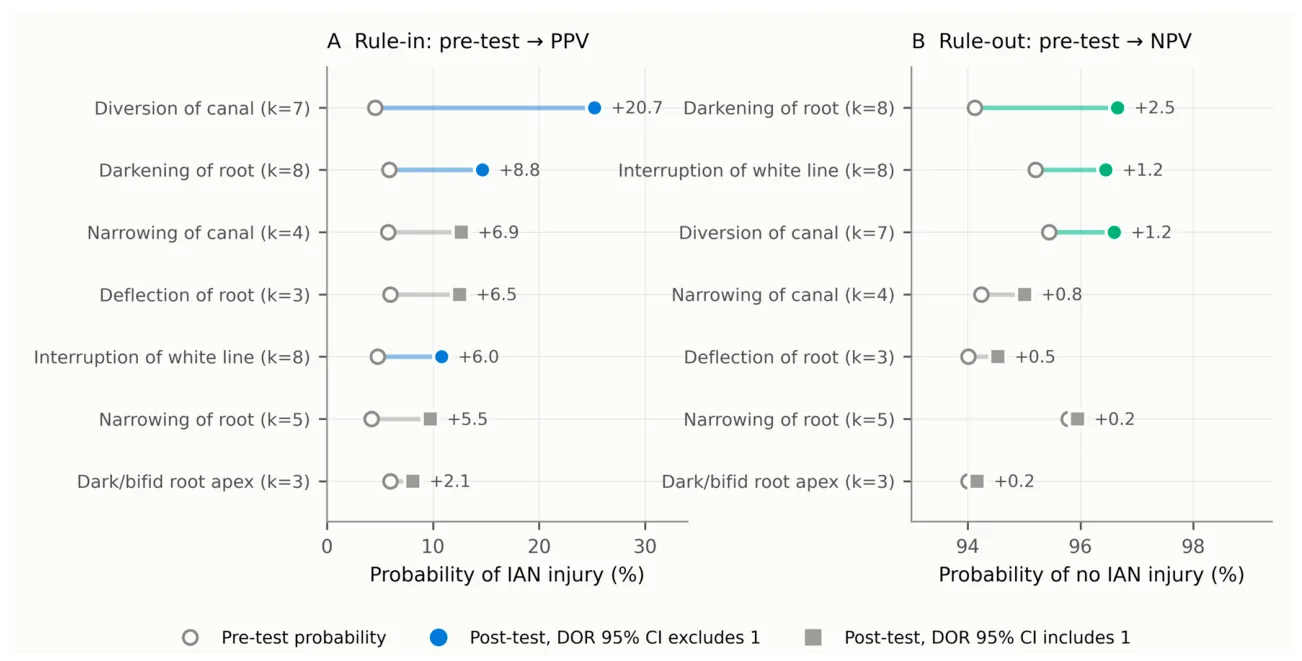
Change from pre-test to post-test probability for each panoramic radiographic sign. (**A**) Rule-in: prevalence of inferior alveolar nerve injury compared with the positive predictive value when the sign is present. (**B**) Rule-out: pre-test probability of no injury compared with the negative predictive value when the sign is absent. Open markers denote pre-test probability, filled markers post-test probability and the annotation gives the absolute change in percentage points. Signs whose diagnostic odds ratio confidence interval includes 1 are shown in grey.

That high negative predictive value largely reflects the low event rate rather than discriminative performance. The pooled prevalence of postoperative IAN injury in the contributing studies was between 4.2% and 6.0%, so the pre-test probability that a given patient will not sustain an injury was already in the range from 94.0% to 95.8% before any radiograph was read. The absolute gain conferred by the absence of a sign is therefore small: 2.5 percentage points for darkening of the root, 1.2 for interruption of the white line and for diversion of the canal and 0.2 to 0.8 for the remaining four signs. Table 4 reports this incremental change alongside each predictive value, and Figure 4 shows it directly.

The corresponding gain on the rule-in side is larger in relative terms. Diversion of the canal raises the probability of injury from 4.6% to 25.2%, an absolute increase of 20.7 percentage points, and darkening of the root raises it from 5.9% to 14.7%. Panoramic signs are therefore more informative when present than when absent, which is the reverse of the interpretation offered in the submitted version of this manuscript.

**Table 6:** Pooled prevalence and predictive values of panoramic radiographic signs for postoperative inferior alveolar nerve injury, with the absolute change from the corresponding pre-test probability in percentage points (pp). k, number of contributing studies; PPV, positive predictive value; NPV, negative predictive value.

Radiographic Sign	k	Prevalence, % (95% CI)	PPV, % (95% CI)	Change in PPV, pp	Pre-Test P (No Injury), %	NPV, % (95% CI)	Change in NPV, pp
Darkening of the root	8	5.9 (5.0–6.9)	14.7 (11.9–17.9)	+8.8	94.1	96.7 (95.7–97.4)	+2.5
Interruption of the white line	8	4.8 (4.0–5.7)	10.8 (8.2–14.1)	+6.0	95.2	96.4 (95.6–97.2)	+1.2
Diversion of the canal	7	4.6 (3.8–5.5)	25.2 (18.2–33.9)	+20.7	95.4	96.6 (95.7–97.3)	+1.2
Narrowing of the canal	4	5.8 (4.3–7.7)	12.7 (6.8–22.4)	+6.9	94.2	95.0 (93.0–96.4)	+0.8
Narrowing of the root	5	4.2 (3.3–5.4)	9.8 (3.9–22.5)	+5.5	95.8	95.9 (94.8–96.9)	+0.2
Deflection of the root	3	6.0 (4.3–8.3)	12.5 (5.5–26.1)	+6.5	94.0	94.5 (92.2–96.2)	+0.5
Dark and bifid root apex	3	6.0 (4.3–8.3)	8.1 (2.8–21.3)	+2.1	94.0	94.2 (91.7–95.9)	+0.2

The prevalence from 4.2% to 6.0% observed in the contributing studies is not the prevalence a given clinic should assume. Several contributing studies enrolled only patients already judged radiographically high risk, which raises the observed event rate; a series of routine extractions of shallowly impacted teeth would lower it. Because predictive values move with prevalence while likelihood ratios do not, post-test probabilities were recomputed from the pooled likelihood ratios across baseline risks from 2% to 15% (Table 7). The clinical reading is stable across that range. The rule-in gain from diversion of the canal is substantial at every baseline risk, taking from 2% to 10.4%, 5% to 23.0% and 15% to 50.0%, whereas the rule-out gain from its absence is small at every baseline risk, moving 5% only to 3.9% and 15% only to 12.0%. A clinic whose own injury rate differs materially from the pooled figure should read Table 7 rather than Table 6.

**Table 7:** Post-test probability of postoperative inferior alveolar nerve injury when each panoramic sign is present or absent, across three baseline risks: 2% (routine extraction), 5% (approximately the pooled prevalence in the contributing studies) and 15% (a population already selected as high risk). Values are derived from the pooled likelihood ratios and are therefore independent of the prevalence observed in the contributing studies. LR+, positive likelihood ratio; LR−, negative likelihood ratio.

Radiographic Sign	LR+	LR−	2%: Sign Present	2%: Sign Absent	5%: Sign Present	5%: Sign Absent	15%: Sign Present	15%: Sign Absent
Darkening of the root	2.32	0.60	4.5%	1.2%	10.9%	3.1%	29.1%	9.6%
Interruption of the white line	2.35	0.75	4.6%	1.5%	11.0%	3.8%	29.3%	11.7%
Diversion of the canal	5.66	0.77	10.4%	1.5%	23.0%	3.9%	50.0%	12.0%
Narrowing of the canal	1.80	0.93	3.5%	1.9%	8.7%	4.6%	24.1%	14.0%
Narrowing of the root	4.60	0.90	8.6%	1.8%	19.5%	4.5%	44.8%	13.7%
Deflection of the root	2.97	0.88	5.7%	1.8%	13.5%	4.4%	34.4%	13.4%
Dark and bifid root apex	2.34	0.91	4.6%	1.8%	11.0%	4.6%	29.2%	13.8%

### 3.6. Intraoperative Nerve Exposure and Radiographic Canal Contact

One study, Sedaghatfar et al. (2005) [50], reported two × two data against intraoperative nerve exposure rather than postoperative injury [7,44]. Its estimates are reported separately and were not pooled with the primary analysis. In that study, interruption of the white line had a sensitivity of 0.75 (95% CI 0.54–0.88) and specificity of 0.66 (95% CI 0.62–0.71) for nerve exposure, and diversion of the canal a sensitivity of 0.42 and specificity of 0.89. Sensitivity against exposure is markedly higher than the pooled sensitivity of the same signs against postoperative injury, which is the expected direction: exposure of the nerve is a more frequent event than injury to it, and a radiographic sign of proximity predicts proximity better than it predicts harm.

Ten included studies compared panoramic signs with CBCT or medical CT findings. These consistently report that darkening of the root and interruption of the white line are associated with CBCT-confirmed loss of cortication or direct canal contact [12,13,28,38,55]. Ruiz-Roca et al. (2024) [58], for example, reported a sensitivity of 81.2% and specificity of 33.2% for panoramic prediction of CBCT-confirmed contact [55]. These estimates are not combined with the primary analysis, and they should not be read as accuracy for predicting nerve injury: they describe how well a two-dimensional radiographic sign predicts a three-dimensional anatomical finding, which is a different question.

### 3.7. Risk of Bias

Domain-level QUADAS-2 judgements for all ten studies contributing two × two accuracy data are presented in Table 8, and the source and rationale for every judgement in Supplementary Table S7. No contributing study was at low risk of bias across all four domains. Three recurrent concerns were identified, of which the third is the most consequential. Patient selection was compromised in the three studies that restricted enrolment to patients already considered at high risk on panoramic imaging (Gallesio et al. 2010 [26], Hasegawa et al. 2013 [24], Harada et al. 2015 [27]), which conditions enrolment on the index test, enriches the sample for risk and raises the observed prevalence on which the predictive values depend. The index-test domain was at unclear risk in six studies because blinding of radiographic interpretation to the subsequent surgical or clinical findings was not reported; only Hasegawa et al. (2013) [24] state that panoramic and CT findings were read independently and blinded to postoperative nerve status.

These domain-level findings materially qualify the pooled estimates and are the principal reason that certainty in the evidence is rated low. Because no contributing study was at low risk of bias across all four domains, a sensitivity analysis restricted to studies at overall low risk was not feasible; the four sensitivity analyses described in Section 2.9 were performed instead and are reported in Section 3.9.

The reference-standard domain was the principal source of bias, judged at high risk in seven of the ten studies and at low risk in none, and the specific concern differs between them. In Ghaeminia et al. (2015) [16], nerve status was self-reported by trial participants who were necessarily unblinded to the imaging they had received. In Rood and Shehab (1990) [21], the method of postoperative sensory assessment is neither standardised nor described, and the study combines a retrospective and a prospective survey. In Harada et al. (2015) [27], paraesthesia was recorded as reported at a single review one week after surgery, with no neurosensory protocol described. In Sedaghatfar et al. (2005) [50], the reference standard is intraoperative visualisation recorded by the operating surgeon, who had already seen the radiograph, and the target condition is exposure rather than injury. In Huang et al. (2015) [25], Gomes et al. (2008) [22] and Gallesio et al. (2010) [26], a clinical neurosensory examination was performed but blinding of the assessor to the panoramic findings is not reported. The three remaining studies were judged unclear rather than high: Leung and Cheung (2011) [23] and Szalma et al. (2010) [10] do not describe how nerve status was ascertained, and Hasegawa et al. (2013) [24] used pin-prick and light-touch testing with follow-up to six months but does not state whether the assessor was blinded. The common thread is that in nine of ten studies, the person who judged whether the nerve was injured may have known what the radiograph showed, which inflates the apparent association between sign and outcome in an unknown direction of magnitude but a predictable direction of sign.

**Table 8:** QUADAS-2 risk of bias and applicability judgements at domain level for the ten studies contributing two × two data. L, low risk; H, high risk; U, unclear.

Study (Year) [Ref]	Patient Selection	Index Test	Reference Standard	Flow and Timing	Applicability: Patient Selection	Applicability: Index Test	Applicability: Reference Standard
Huang 2015 [25]	Low	Low	High	Low	Low	Low	Low
Gomes 2008 [22]	Unclear	Unclear	High	Low	Low	Low	Low
Rood 1990 [21]	Unclear	Unclear	High	Unclear	Low	Low	Low
Leung 2011 [23]	Low	Unclear	Unclear	Low	Low	Low	Low
Ghaeminia 2015 [16]	Low	Low	High	Low	Low	Low	Low
Gallesio 2010 [26]	High	Unclear	High	Low	Low	Low	Low
Sedaghatfar 2005 [50]	Low	Unclear	High	Low	Low	Low	High
Hasegawa 2013 [24]	High	Low	Unclear	Low	High	Low	Low
Harada 2015 [27]	High	Unclear	High	Unclear	High	Low	Unclear
Szalma 2010 [10]	High	Unclear	Unclear	High	High	Low	Low

Leave-one-out analyses were performed for every sign, and the range of the diagnostic odds ratio and of both likelihood ratios across iterations is reported in Supplementary Table S9. For darkening of the root, the pooled diagnostic odds ratio ranged from 3.25 to 5.32 and remained statistically significant after removal of any single study, with the positive likelihood ratio between 2.03 and 2.71 and the negative likelihood ratio between 0.53 and 0.65. For diversion of the canal, the diagnostic odds ratio ranged from 5.86 to 13.33 and likewise remained significant throughout, with the positive likelihood ratio between 4.20 and 7.56 and the negative likelihood ratio between 0.72 and 0.82. The association for interruption of the white line was not stable: the confidence interval crossed 1 when any one of five studies was omitted, the diagnostic odds ratio ranged from 2.65 to 6.01, and the positive likelihood ratio fell as low as 1.78. Two points follow from reporting the likelihood ratios rather than the odds ratio alone. Even at the top of its leave-one-out range, darkening of the root has a positive likelihood ratio below 3, which is a modest shift in probability rather than a decisive one; and the negative likelihood ratio never falls below 0.53 for any sign, so no sign approaches the value of about 0.1 conventionally regarded as useful for ruling a condition out. Statistical significance and clinical usefulness are therefore not the same claim, and only the second is at issue at the chairside.

The four randomised trials comparing CBCT with panoramic radiography addressed clinical utility rather than diagnostic accuracy and were therefore appraised separately using RoB 2 (Table 9). Three trials raised some concerns, principally because blinding of participants and surgeons was not feasible or was not reported while sensory disturbance was an important outcome. Petersen et al. (2016) [18] used central randomisation with blinding of participants and assessors and was judged at low risk across all domains. These trials contributed no data to the pooled diagnostic-accuracy estimates [18].

**Table 9:** RoB 2 assessment of the four randomised trials comparing cone-beam computed tomography with panoramic radiography for postoperative outcomes. These trials inform clinical utility and contribute no data to the pooled accuracy estimates.

Trial (Year) [Ref]	Primary Outcome	Overall RoB 2 Judgement	Principal Concern
Guerrero et al. (2014) [17]	Postoperative complications	Some concerns	Participants and surgeons not blinded
Ghaeminia et al. (2015) [16]	IAN injury (self-reported)	Some concerns	Participant unblinding for a subjective outcome
Guerrero et al. (2012) [49]	IAN sensory testing	Some concerns	Subjective outcome components; participants unblinded
Petersen et al. (2016) [18]	Neurosensory disturbance	Low risk	None substantial; central randomisation, blinded assessment

### 3.8. Clinical Utility

Clinical utility is reported separately from accuracy because the two questions have different designs and different answers. The evidence divides into two groups that point in different directions.

Studies of decision making report that three-dimensional imaging changes the operative plan. Yüce et al. (2021) [45] found that CBCT changed the treatment decision in 85 of 150 high-risk patients, most often from extraction to coronectomy [45]. Susarla and Dodson (2007) [53] reported that CT reclassified 62% of panoramically high-risk cases as low risk [53]. Korkmaz et al. (2017) [43] reported a lower rate of temporary nerve injury with CBCT in high-risk cases (4.2% versus 16.4%), with no difference in permanent injury [43].

Randomised trials of patient outcome report no benefit. Ghaeminia et al. (2015) [16], Guerrero et al. (2014) [17], Guerrero et al. (2012) [49] and Petersen et al. (2016) [18] each found no significant reduction in postoperative neurosensory disturbance with CBCT compared with panoramic radiography [16,17,18,49]. The meta-analysis by Clé-Ovejero et al. (2017) [59] reached the same conclusion [59].

The reconciliation is that CBCT changes what surgeons plan without yet being shown to change what happens to patients. The trials were individually underpowered for an outcome with an event rate below 5%, so absence of demonstrated benefit is not demonstrated absence of benefit. The evidence supports selective use of CBCT as a reasonable risk-stratified practice; it does not support a claim that panoramic findings improve nerve outcomes, and no such claim is made here.

These two bodies of evidence can be joined to the accuracy estimates in Table 4. Diversion of the canal has a positive likelihood ratio of 5.66, so in a population with a 5% injury rate its presence raises the estimated probability of injury to about 23%, and in the kind of high-risk population in which CBCT is actually considered, with a baseline nearer 15%, to about 50% (Table 7). Using diversion of the canal as the trigger for a scan would select roughly one patient in twenty, since the sign was present in 115 of 2172 units in the pooled data. Against that has to be set the additional radiation of a CBCT examination, several times that of a panoramic radiograph even with a small field of view, and the finding that the randomised trials did not show a reduction in neurosensory disturbance in the patients who were scanned. What CBCT has been shown to change is the operative plan, most often towards coronectomy. A change in plan is not in itself a benefit to the patient, and this review does not treat it as one. The case for selective CBCT after a high-risk panoramic finding rests on better anatomical information for a decision the surgeon has to make in any event, not on demonstrated protection of the nerve.

Several included studies report that accuracy improves when two or more signs are present together [3,12,51]. No study reported two × two data for combinations of signs, so this could not be quantified in the present analysis; it is reported as a consistent observation across primary studies and as a priority for future work.

### 3.9. Sensitivity Analyses

Restricting the analysis to the four studies that used the patient as the unit of analysis (Huang et al. 2015 [25], Gallesio et al. 2010 [26], Harada et al. 2015 [27], Szalma et al. 2010 [10]) left three to four studies and 534 to 843 units per sign. Point estimates were of the same order as in the primary analysis but the intervals were much wider, and none of the three signs with significant pooled associations remained significant: darkening of the root 2.40 (95% CI 0.78–7.36), diversion of the canal 4.95 (0.75–32.59) and interruption of the white line 1.10 (0.11–11.37). Narrowing of the canal was the only sign whose interval excluded 1 in this analysis, at 3.10 (1.06–9.01), and that is the same three-study comparison as the reconstruction below in which the Leung and Cheung row is omitted. This analysis neither confirms nor contradicts the primary estimates, because removing the five tooth-level studies removes about two thirds of the data. Its usable conclusion is a negative one: the literature does not contain enough patient-level data to estimate the accuracy of any panoramic sign without relying on tooth-level studies whose clustering cannot be quantified. That is a limitation of the evidence base rather than of this synthesis, and it is the reason the confidence intervals in Table 4 should be read as a lower bound on the true uncertainty.

Excluding Szalma et al. (2010) [10], the one contributing study with a case–control sampling design affected only darkening of the root, the single sign to which it contributes data. The pooled diagnostic odds ratio moved from 3.92 (95% CI 1.97–7.80) to 3.77 (1.57–9.08) and pooled sensitivity from 0.54 to 0.50. No estimate for any other sign and no conclusion of the review changes.

The narrowing-of-the-canal row from Leung and Cheung (2011) [23] is internally inconsistent: the four extracted cells sum to 187 against a reported denominator of 178. Four handlings are arithmetically admissible and they do not agree. Reducing the true-negative count from 169 to 160 gives a pooled diagnostic odds ratio of 2.67 (95% CI 0.98–7.28); reducing the false-negative count to zero, or the false-positive count to zero, gives 3.48 (1.24–9.77) in either case; omitting the row gives 3.10 (1.06–9.01). Three of the four therefore yield an interval excluding 1 and one does not. The most conservative reconstruction is the one reported in Table 4 and in the abstract, and the full set is in Supplementary Table S10. Narrowing of the canal is treated throughout as a sign of unproven value, and the reason is stated plainly rather than buried: on the present evidence, its statistical significance turns on how a single unverifiable row is reconstructed, and only the source data, which would have to be obtained from the authors of that study, could settle it.

## 4. Discussion

This systematic review and meta-analysis evaluated the diagnostic accuracy of individual panoramic radiographic signs for predicting postoperative inferior alveolar nerve (IAN) injury following mandibular third molar surgery. Three findings are particularly important. First, darkening of the root and diversion of the canal showed associations with postoperative IAN injury that remained statistically significant in leave-one-out analyses, whereas the association for interruption of the white line was less stable and should therefore be regarded as suggestive rather than established. The remaining four signs did not show statistically conclusive associations with postoperative injury. Second, sensitivity was low for all seven signs, indicating that the absence of a panoramic risk sign does not exclude postoperative IAN injury. Third, the high negative predictive values observed across signs largely reflect the low underlying prevalence of injury; consequently, a negative finding provides only a small incremental increase in the probability of remaining free from injury. This interpretation is consistent with previous evidence showing that the incremental rule-out value of negative panoramic signs is limited [2,3].

The clinical implications are therefore asymmetric. Diversion of the canal produced the largest increase in estimated risk, with the probability of postoperative injury increasing from approximately 5% before consideration of the sign to approximately 25% when the sign was present. In contrast, its absence provided relatively little additional reassurance. Panoramic radiography is therefore better regarded as a first-line risk-stratification tool than as a diagnostic test capable of ruling postoperative IAN injury in or out. A high-risk panoramic finding may reasonably prompt consideration of additional assessment, but the present meta-analysis does not establish a specific probability threshold at which CBCT, coronectomy or specialist referral becomes necessary.

The present estimates differ from some previous reviews principally because this synthesis distinguishes the target conditions used as reference standards. Studies evaluating postoperative neurosensory injury were retained as the primary diagnostic-accuracy analysis, whereas intraoperative nerve exposure and CBCT-defined tooth–canal contact were considered separately. These outcomes are clinically related but are not interchangeable: anatomical contact or intraoperative exposure of the nerve does not necessarily result in postoperative neurosensory injury, and combining them obscures what a pooled accuracy figure is an estimate of. Previous reviews similarly found limited sensitivity despite relatively high specificity for several panoramic signs [2,3]. The present findings therefore refine rather than overturn the earlier evidence, by applying an explicit distinction between postoperative injury and anatomical proximity and by reporting what each pooled estimate is conditional on.

Comparison with cone-beam computed tomography requires the same distinction between anatomical information, prediction of injury and improvement in patient outcomes. CBCT identifies direct contact or loss of cortical separation that a two-dimensional image cannot characterise, and panoramic signs, particularly darkening of the root and interruption of the white line, are associated with those CBCT findings [16,41,59]. It may also alter operative planning in selected high-risk cases, including the choice between complete removal and coronectomy [45,53]. None of that demonstrates improved postoperative outcomes. The randomised trials included here did not consistently show a reduction in neurosensory disturbance with routine CBCT compared with panoramic assessment alone [16,17,18,49], and given the low incidence of injury, they had limited power to detect a modest difference. The correct reading is an absence of demonstrated outcome benefit from routine CBCT rather than evidence that CBCT confers none, and it does not license a claim that panoramic findings improve nerve outcomes.

The findings also highlight the importance of distinguishing statistical association from clinical prediction. Even where a panoramic sign is significantly associated with postoperative injury, its usefulness depends on both its likelihood ratios and the underlying prevalence of injury. Diversion of the canal produced the greatest increase in post-test probability in the present analysis, whereas darkening of the root produced a more modest increase. Interruption of the white line showed an overall association, but this finding was sensitive to the removal of individual studies and should therefore be interpreted cautiously. Conversely, the high negative predictive values observed for several signs should not be interpreted as strong rule-out performance because the probability of remaining injury free was already high before the panoramic finding was considered [2].

A concrete example makes the distinction usable. Two patients attend for removal of a deeply impacted lower third molar in a practice whose own rate of postoperative sensory disturbance is about 5%. In the first, the panoramic radiograph shows diversion of the canal; the positive likelihood ratio of 5.66 raises that patient’s estimated risk to roughly 23%, which is a different conversation about consent and a defensible reason to obtain three-dimensional imaging or to discuss coronectomy. In the second, no high-risk sign is present; the negative likelihood ratio of 0.77 lowers the estimated risk only from 5% to about 3.9%. The second patient has not been shown to be safe, because the radiograph has barely moved the number. This asymmetry, and not the statistical significance of the underlying association, is what determines whether a sign is worth acting on.

The methodological limitations of the evidence base should also temper interpretation of the pooled estimates. No study contributing two × two data was judged to be at low risk of bias across all four QUADAS-2 domains, and the reference-standard domain was at high risk of bias in seven of ten studies. Neurosensory outcomes were not always assessed using standardised methods, and blinding of outcome assessment to the panoramic findings was frequently absent or insufficiently reported. Patient selection also varied, with some studies enrolling populations already considered radiographically high risk. Such selection may affect the observed prevalence of injury and consequently, the predictive values. These limitations contribute to uncertainty around the magnitude of the pooled effects and argue against treating any single panoramic sign as a definitive clinical decision rule.

Taken together, these findings support the use of panoramic radiography primarily for initial risk stratification rather than definitive prediction of postoperative IAN injury. A positive high-risk sign indicates an increased probability of injury rather than certainty that injury will occur, while the absence of such signs does not eliminate risk. Decisions regarding additional three-dimensional imaging should therefore incorporate the panoramic findings together with the wider clinical and surgical context. This interpretation is consistent with the limited incremental predictive value of negative panoramic findings [2] and with the absence of a consistently demonstrated reduction in postoperative IAN injury from routine CBCT [16,17,18,49].

### 4.1. Clinical Implications

The clinical value of panoramic radiography lies primarily in risk stratification rather than in excluding postoperative IAN injury. The absence of the three signs showing the strongest associations provides only limited additional reassurance because the baseline risk of injury is already low. Accordingly, the decision to obtain additional imaging should not be based solely on the absence or presence of an individual panoramic sign but should incorporate the overall clinical and radiographic context. Routine CBCT has not been shown consistently to reduce postoperative IAN injury, although it provides additional three-dimensional anatomical information and may alter surgical planning in selected high-risk cases. This selective approach is consistent with recommendations that CBCT should not be used routinely before MM3 removal but should address a specific clinical question that cannot be adequately resolved by conventional imaging.

Diversion of the canal produced the largest increase in estimated risk, with the probability of postoperative injury increasing from approximately 5% before consideration of the sign to approximately 25% when the sign was present. This substantial increase may reasonably prompt consideration of further three-dimensional assessment, alternative surgical strategies such as coronectomy or specialist referral, depending on the clinical context. Evidence from clinical practice also indicates that CBCT findings can influence the choice between complete removal and coronectomy in selected high-risk cases. Darkening of the root and interruption of the white line were associated with more modest increases in risk and are better interpreted alongside other clinical and surgical factors than as standalone indications for additional imaging. For the purposes of this review, a high-risk panoramic finding means diversion of the canal, which is the only single sign with both a positive likelihood ratio above 5 and an association that survives removal of any individual study. Darkening of the root, with a positive likelihood ratio of 2.32, does not reach that standard on its own. No threshold of post-test probability at which CBCT, coronectomy or specialist referral becomes indicated can be derived from these data and none is proposed: those decisions turn on the depth and angulation of the tooth, the operator’s experience, the patient’s own tolerance of risk and the local availability of three-dimensional imaging. The estimates reported here are context for such decisions rather than rules that determine them.

Four of the seven signs assessed narrowing of the canal, narrowing of the root, deflection of the root and a dark and bifid root apex did not show statistically conclusive associations with postoperative IAN injury in the present meta-analysis. These findings do not establish that these signs have no clinical value; rather, the available evidence is insufficient to support their use as standalone predictors of postoperative injury or as standalone triggers for additional imaging. The traditional panoramic signs should therefore not necessarily be interpreted as having equivalent predictive value.

Preoperative counselling should reflect both the estimated risk and its uncertainty rather than rely on a single panoramic finding or point estimate. A patient without the more informative panoramic signs still faces a small but clinically relevant risk of postoperative sensory disturbance, and a negative panoramic finding does not eliminate that risk. Conversely, a positive high-risk sign indicates increased probability rather than certainty of injury. A selective approach to additional imaging, based on the combined clinical and radiographic assessment, is therefore more consistent with the limited incremental predictive value of panoramic findings and with the principle of avoiding unnecessary radiation exposure.

### 4.2. Strengths and Limitations

The main strength of this review is that the quantitative synthesis is restricted to a single, clinically meaningful target condition. Every pooled estimate answers the question a surgeon actually asks: how well does this radiographic sign predict that this patient will have altered sensation afterwards rather than blending it with anatomical proximity. The analysis is fully specified and reproducible, the contribution of each study to each estimate is stated, and the two data errors identified during checking are reported rather than corrected silently.

The limitations are substantial and constrain what can be concluded. Heterogeneity was high for every sign, with I^2^ for specificity between 91% and 98%, and the pooled estimates should not be transferred to an individual clinic without allowance for that. No study was at low risk of bias across all four QUADAS-2 domains, and blinding of radiographic interpretation to outcome was frequently absent or unreported. Several studies enrolled only patients already identified as high risk, which may increase the observed prevalence of injury and affect predictive values. Most studies were retrospective. Only 10 of the 37 included studies contributed two × two data, and four of the seven signs rest on three to five studies each. Definitions of the signs themselves varied, as did the interval and method of neurosensory assessment, so that the same label does not always denote the same finding. Some studies analysed teeth rather than patients without accounting for clustering. The English-language restriction applied at eligibility assessment, and the absence of a systematic grey-literature search beyond Google Scholar may have excluded eligible evidence, although the likely direction of that bias runs against rather than towards the conclusions drawn here (Section 2.4). Certainty in the pooled estimates is rated low. Two further limitations deserve emphasis because they bound what this review can be used for. The first is that clinical decisions rest on the whole radiograph rather than on one feature; several included studies report that accuracy improves when two or more signs are present together, yet no study reported two × two data for any defined combination. The accuracy of combined signs the quantity a surgeon actually reads off a radiograph could therefore not be estimated at all, and nothing here should be taken as an estimate of it. The second is that the pooled confidence intervals are narrower than the data support, because clustering in the five tooth-level studies could not be quantified and no variance inflation was applied.

### 4.3. Future Research

Three priorities follow directly from these limitations. Standardised operational definitions of the panoramic signs, with published reference images and reported inter-observer agreement, would remove one of the largest sources of heterogeneity. Prospective diagnostic accuracy studies with blinded radiographic interpretation, a pre-specified neurosensory assessment protocol and a defined follow-up interval are needed; the present evidence base contains very few. Because combinations of signs appear to outperform single signs but have never been reported with extractable two × two data, studies should report accuracy for defined combinations as well as for individual features. Machine-learning models trained on panoramic images are a promising direction and should be evaluated against the same target condition and reference standard used here, so that their performance can be compared with the estimates in Table 6 [57,60].

## 5. Conclusions

Three target conditions have to be kept apart in this literature, and this review keeps them apart: postoperative neurosensory injury, intraoperative exposure of the nerve and radiographic contact between root and canal on CBCT. Only the first is an event that matters to a patient, and the conclusions below concern it alone. Panoramic radiography has limited standalone accuracy for predicting inferior alveolar nerve injury after mandibular third molar surgery. Of the seven radiographic signs assessed against postoperative neurosensory outcomes, darkening of the root, interruption of the white line and diversion of the canal showed statistically significant pooled associations with postoperative IAN injury; however, the association for interruption of the white line was less stable in leave-one-out analyses and should therefore be interpreted cautiously, whereas the associations for darkening of the root and diversion of the canal remained stable. The remaining four signs did not show statistically conclusive associations with postoperative injury. Sensitivity was low for all seven signs, so the absence of these features does not exclude injury. The high negative predictive values observed largely reflect the low background event rate and provide only limited incremental reassurance beyond the pre-test probability.

Diversion of the canal was the most informative individual finding, increasing the estimated probability of postoperative injury from approximately 5% to approximately 25%. Panoramic radiography is therefore best regarded as a first-line risk-stratification tool rather than a definitive diagnostic test. A high-risk finding, particularly diversion of the canal, may reasonably prompt consideration of additional three-dimensional imaging, alternative surgical strategies or specialist referral, depending on the overall clinical context. Conversely, the absence of high-risk panoramic findings provides only limited additional reassurance and should not be interpreted as excluding postoperative IAN injury. Substantial between-study heterogeneity and methodological limitations, including the absence of any contributing study at low risk of bias across all four QUADAS-2 domains, mean that the pooled estimates should be interpreted cautiously and regarded as provisional. Two limits should travel with these figures. The accuracy of combinations of signs, which is what a surgeon actually reads off a radiograph, could not be estimated because no study reported the data needed to do so. And the pooled estimates rest on ten studies, none at low risk of bias across all four QUADAS-2 domains and seven at high risk in the reference-standard domain, with heterogeneity in specificity between 91% and 98% and confidence intervals that clustering makes narrower than the data support. They are provisional, and on this evidence, the absence of a high-risk panoramic sign does not exclude postoperative inferior alveolar nerve injury.

## Data Availability

All data supporting the findings of this review are contained within the article and its Supplementary Materials. The extracted two × two dataset, the analysis code used to produce Table 4, Table 5, Table 6 and Table 7 and Figure 2, Figure 3 and Figure 4, and and the record of the data corrections described in Supplementary Table S5 are provided as Supplementary Material.

## Supplementary Materials

The following supporting information can be downloaded at https://www.mdpi.com/article/10.3390/jcm15218315/s1: Table S1: PRISMA 2020 checklist. Table S2: PRISMA-DTA checklist. Table S3: Reports excluded at full-text assessment, with reasons. Table S4: Search strategy for each source, with records retrieved. Table S5: Data audit, including the handling of the two internally inconsistent extracted rows and the unstratified analysis. Table S6: Extended characteristics of all 37 included studies. Table S7: Source and rationale for every QUADAS-2 domain judgement. Table S8: Operational reference-standard definitions for the ten studies contributing two × two data. Table S9: Leave-one-out analyses with diagnostic odds ratio and likelihood-ratio ranges. Table S10: Arithmetically admissible reconstructions of the Leung and Cheung (2011) [23] narrowing-of-the-canal row.
