# Supplementary material for: Diagnostic Accuracy of Panoramic Radiographic Signs for Predicting Inferior Alveolar Nerve Injury Following Mandibular Third Molar Surgery: A Systematic Review and Meta-Analysis
Source: J Clin Med. 2026 Oct 26;15(21):8315. doi: 10.3390/jcm15218315 (PMC13764449; doi:10.3390/jcm15218315)
Supplement: Supplementary file 1 [file jcm-15-08315-s001.zip › jcm-4599066-supplementary.pdf]

Diagnostic Accuracy of Panoramic Radiographic Signs for Predicting Inferior Alveolar Nerve Injury Following Mandibular Third Molar Surgery: A Systematic Review and Meta-Analysis

Supplementary Materials — Tables S1 to S10

Ahmad Othman and Albarra Alolayan

Contents

Table S1 PRISMA 2020 checklist
Table S2 PRISMA-DTA checklist
Table S3 Reports excluded at full-text assessment, with reasons
Table S4 Search strategy for each source, with records retrieved
Table S5 Data audit, including the handling of the two internally inconsistent extracted rows and the un-stratified analysis
Table S6 Extended characteristics of all 37 included studies
Table S7 Source and rationale for every QUADAS-2 domain judgement
Table S8 Operational reference-standard definitions for the ten studies contributing two × two data
Table S9 Leave-one-out analyses with diagnostic odds ratio and likelihood-ratio ranges
Table S10 Arithmetically admissible reconstructions of the Leung and Cheung (2011) [23] narrow-ing-of-the-canal row

Table S1. PRISMA 2020 checklist

Item numbering follows Page et al. (2021). The final column gives the location in the manuscript where each item is reported.

Item	Section	Checklist item	Reported in
1	Title	Identify the report as a systematic review	Title page
2	Abstract	Structured summary	Abstract
3	Rationale	Describe the rationale in the context of existing knowledge	Section 1, paragraphs 1–6
4	Objectives	Explicit statement of objectives or questions	Section 1, final paragraph; Section 2.2
5	Eligibility criteria	Inclusion and exclusion criteria and grouping for synthesis	Section 2.5
6	Information sources	All databases, registers and other sources; date last searched	Section 2.4 (five databases; final search 24 June 2025)
7	Search strategy	Full search strategies for all sources, including filters	Section 2.4 (complete strings for all five databases); Supplementary Table S4
8	Selection process	Methods, number of reviewers, independence, automation tools	Section 2.7

Item	Section	Checklist item	Reported in
9	Data collection process	Methods, number of reviewers, independence, automation tools	Section 2.7
10a	Data items — outcomes	All outcomes sought; domains and definitions	Sections 2.3 and 2.7 (TP/TN/FP/FN definitions)
10b	Data items — other variables	All other variables sought	Sections 2.6 and 2.7; Table 1
11	Study risk of bias assessment	Tools used, number of reviewers, independence	Section 2.8 (QUADAS-2 primary; RoB 2 for randomised trials)
12	Effect measures	Effect measures used in synthesis or presentation	Section 2.9 (sensitivity, specificity, LR+, LR−, DOR, SROC AUC, PPV, NPV)
13a	Synthesis — eligibility	Process for deciding which studies were eligible for each synthesis	Sections 2.9 and 3.3
13b	Synthesis — data preparation	Handling of missing data and data conversions	Sections 2.7 and 2.9 (continuity correction; consistency checks)
13c	Synthesis — tabulation	Methods to tabulate or visually display results	Tables 2–4; Figures 2–4
13d	Synthesis — methods	Methods used to synthesise results; model, method and software	Section 2.9 (DerSimonian–Laird; Python 3.11, NumPy 2.4, SciPy 1.17)
13e	Synthesis — heterogeneity	Methods to explore causes of heterogeneity	Sections 2.9 and 3.4 (I^2, stratification by target condition)
13f	Synthesis — sensitivity	Sensitivity analyses to assess robustness	Sections 2.9 and 3.7 (three sensitivity analyses)
14	Reporting bias assessment	Methods to assess risk of bias due to missing results	Section 2.9 — formal assessment not undertaken; reason stated
15	Certainty assessment	Methods to assess certainty in the body of evidence	Sections 3.7 and 4.2 — certainty rated low
16a	Study selection	Numbers screened, assessed and included, with a flow diagram	Section 3.1; Figure 1
16b	Excluded studies	Studies that appeared to meet criteria but were excluded, with reasons	Figure 1; Supplementary Table S3
17	Study characteristics	Cite each included study and present its characteristics	Table 1; Supplementary Table S6
18	Risk of bias in	Risk of bias assessments for each included study	Table 5; Supplementary Table S7; Table 6

Item	Section	Checklist item	Reported in
	studies		
19	Results of individual studies	Summary statistics and effect estimates for each study	Table 2; Supplementary Table S5
20a	Results of syntheses — studies	Characteristics and risk of bias among contributing studies	Sections 3.3 and 3.7
20b	Results of syntheses — estimates	Summary estimates and precision; direction of effect	Section 3.4; Tables 3 and 4; Figures 2–4
20c	Results of syntheses — heterogeneity	Causes of heterogeneity investigated	Sections 3.4 and 3.7
20d	Results of syntheses — sensitivity	Results of sensitivity analyses	Section 3.7
21	Reporting biases	Risk of bias due to missing results	Section 2.9; Section 4.2
22	Certainty of evidence	Certainty for each outcome assessed	Sections 3.7 and 4.2
23a	Discussion — interpretation	General interpretation in the context of other evidence	Section 4, opening
23b	Discussion — limitations of evidence	Limitations of the evidence included	Section 4.2
23c	Discussion — limitations of processes	Limitations of the review processes used	Section 4.2
23d	Discussion — implications	Implications for practice, policy and future research	Sections 4.1 and 4.3
24a	Registration	Registration name and number	Section 2.1 (PROSPERO CRD42020214906)
24b	Protocol	Where the protocol can be accessed	Section 2.1
24c	Amendments	Amendments to information provided at registration	Section 2.1 — three amendments declared
25	Support	Sources of financial or non-financial support	Funding statement
26	Competing interests	Competing interests of review authors	Conflicts of Interest
27	Availability of data and code	Availability of data, code and other materials	Data Availability Statement; Supplementary File S5

Table S2. PRISMA-DTA checklist

Item numbering follows McInnes et al. (2018). Items specific to diagnostic test accuracy reviews.

Item	Section	Checklist item	Reported in
1	Title	Identify the report as a systematic review of diagnostic test accuracy	Title page
2	Abstract	Structured summary including index test, reference standard and target condition	Abstract
3	Rationale	Rationale in the context of what is already known	Section 1
4	Objectives	Objectives stated with reference to participants, index test and target condition	Section 1, final paragraph; Section 2.2 (PIRT framing)
5	Protocol and registration	Registration and protocol availability	Section 2.1
6	Eligibility criteria	Study characteristics and report characteristics used as criteria	Section 2.5
7	Information sources	Sources searched with dates of coverage and last search	Section 2.4
8	Search	Full electronic search strategy for at least one database, repeatable	Section 2.4 — full strategy given for all five databases
9	Study selection	Process for selecting studies	Sections 2.7 and 3.1
10	Data collection process	Method of data extraction and any processes for obtaining data from investigators	Section 2.7
11	Definitions for data extraction	Definition of index test, target condition and reference standard	Sections 2.3 and 2.6 — three target conditions defined separately
12	Risk of bias and applicability	Methods used and how information was to be used in synthesis	Section 2.8; Table 5
13	Diagnostic accuracy measures	Primary and alternative measures of accuracy	Section 2.9
14	Synthesis of results	Methods of handling data, combining results and describing variability	Section 2.9
15	Meta-bias	Assessment of publication bias and selective reporting within studies	Section 2.9 — not formally assessed; reason given
16	Additional analyses	Methods of additional analyses and whether pre-specified	Section 2.9 — three sensitivity analyses; stratification declared post hoc

Item	Section	Checklist item	Reported in
17	Study selection	Numbers of studies screened, assessed and included, with reasons for exclusion	Section 3.1; Figure 1
18	Study characteristics	Characteristics of included studies, including target condition and reference standard	Table 1; Supplementary Table S6
19	Risk of bias and applicability	Risk of bias and applicability concerns for each study	Table 5; Supplementary Table S7
20	Results of individual studies	Two-by-two data for each study, by target condition	Table 2; Supplementary Table S5
21	Synthesis of results	Pooled estimates with confidence intervals and measures of consistency	Section 3.4; Tables 3 and 4; Figures 2–4
22	Meta-bias	Results of any assessment of bias across studies	Section 2.9; Section 4.2
23	Additional analyses	Results of additional analyses	Section 3.7
24	Summary of evidence	Summary of main findings including strength of evidence	Section 4, opening; Section 5
25	Limitations	Limitations at study and review level	Section 4.2
26	Conclusions	General interpretation and implications for practice	Sections 4.1 and 5
27	Funding	Sources of funding and role of funders	Funding statement

Table S3. Reports excluded at full-text assessment, with reasons

Seventy reports were excluded after full-text assessment. The reason categories and their counts are reproduced from the screening log below; the full citation for each excluded report is held in the Rayyan screening project and is provided as the accompanying export file.

Reason for exclusion	n	Citations
Study design not matched	56	See accompanying Rayyan export
Intervention not relevant	7	See accompanying Rayyan export
Population not relevant	2	See accompanying Rayyan export
Outcome not matched	1	See accompanying Rayyan export
Intervention and study design not matched	1	See accompanying Rayyan export
PICO not met	1	See accompanying Rayyan export
Intervention, outcomes and study design not matched	1	See accompanying Rayyan export
Population, intervention and study design not matched	1	See accompanying Rayyan export
Total	70	

Table S4. Search strategy for each source, with records retrieved

Part A gives the sources searched, the date of searching and the records retrieved from each, as recorded in the Rayyan project used for de-duplication and screening. Part B reproduces the search strings verbatim.

Part A. Sources and records retrieved

Source	Date searched	Date limits applied	Records retrieved
MEDLINE via PubMed	24 June 2025	1 Jan 1990 – 24 Jun 2025	251
Cochrane Library (CENTRAL)	24 June 2025	No date limit applied at interface	196
Scopus	24 June 2025	PUBYEAR > 1989 and < 2026	126
Embase (Elsevier)	24 June 2025	1990–2025 (publication year)	58
Google Scholar (supplementary)	24 June 2025	1990–2025	112
Reference-list and citation searching (exported via Zotero)	24 June 2025	—	20
Total records identified			763
Duplicate records flagged in Rayyan			229
Duplicates removed before screening			144
Records screened at title and abstract			619

Part B. Search strings, verbatim

Source	Concept	String as run
MEDLINE via PubMed	Concept 1 — Mandibular third molar	("Molar, Third"[Mesh] OR "third molar"[Title/Abstract] OR "third molars"[Title/Abstract] OR "mandibular third molar"[Title/Abstract] OR "mandibular third molars"[Title/Abstract] OR "lower third molar"[Title/Abstract] OR "lower third molars"[Title/Abstract] OR "3rd molar"[Title/Abstract] OR "3rd molars"[Title/Abstract] OR "wisdom tooth"[Title/Abstract] OR "wisdom teeth"[Title/Abstract] OR "wisdom tooth extraction"[Title/Abstract] OR "wisdom teeth extraction"[Title/Abstract] OR "third molar extraction"[Title/Abstract] OR "third molar surgery"[Title/Abstract] OR "M3M"[Title/Abstract] OR "MM3"[Title/Abstract] OR "tooth 38"[Title/Abstract] OR "tooth 48"[Title/Abstract])
	Concept 2 — Panoramic radiography and radiographic signs	("Radiography, Panoramic"[Mesh] OR panoramic[Title/Abstract] OR "panoramic radiograph"[Title/Abstract] OR "panoramic radiographs"[Title/Abstract] OR "panoramic radiography"[Title/Abstract] OR "panoramic imaging"[Title/Abstract] OR orthopantomogram*[Title/Abstract] OR orthopantomograph*[Title/Abstract] OR OPG[Title/Abstract] OR "preoperative radiograph"[Title/Abstract] OR "preoperative radiographs"[Title/Abstract] OR "presurgical radiograph"[Title/Abstract] OR "presurgical radiographs"[Title/Abstract] OR "preoperative imaging"[Title/Abstract] OR "presurgical imaging"[Title/Abstract] OR "preoperative radiography"[Title/Abstract] OR "presurgical radiography"[Title/Abstract] OR "radiographic sign"[Title/Abstract] OR "radiographic signs"[Title/Abstract] OR "radiographic indicator"[Title/Abstract] OR "radiographic indicators"[Title/Abstract] OR "radiographic risk sign"[Title/Abstract] OR "radiographic risk signs"[Title/Abstract] OR "canal diversion"[Title/Abstract] OR "diversion of the canal"[Title/Abstract] OR "interruption of the white line"[Title/Abstract] OR "interruption of white line"[Title/Abstract] OR "darkening of the root"[Title/Abstract] OR "darkening of root"[Title/Abstract] OR "root darkening"[Title/Abstract] OR "root deflection"[Title/Abstract] OR "deflection of the root"[Title/Abstract] OR "root narrowing"[Title/Abstract] OR "narrowing of the root"[Title/Abstract] OR "canal narrowing"[Title/Abstract] OR "narrowing of the canal"[Title/Abstract] OR "dark and bifid apex"[Title/Abstract] OR "bifid root apex"[Title/Abstract])
	Concept 3 — Inferior alveolar nerve injury	("Mandibular Nerve"[Mesh] OR "Paresthesia"[Mesh] OR "inferior alveolar nerve"[Title/Abstract] OR "inferior alveolar nerve injury"[Title/Abstract] OR "inferior alveolar nerve injuries"[Title/Abstract] OR "inferior alveolar nerve damage"[Title/Abstract] OR "inferior alveolar nerve deficit"[Title/Abstract] OR "inferior alveolar nerve dysfunction"[Title/Abstract] OR "IAN injury"[Title/Abstract] OR "IAN injuries"[Title/Abstract] OR "IAN damage"[Title/Abstract] OR "nerve injury"[Title/Abstract] OR "nerve damage"[Title/Abstract] OR paresthesia[Title/Abstract] OR paraesthesia[Title/Abstract] OR hypoesthesia[Title/Abstract] OR hypoaesthesia[Title/Abstract] OR dysesthesia[Title/Abstract] OR dysaesthesia[Title/Abstract] OR "sensory disturbance"[Title/Abstract] OR "sensory disturbances"[Title/Abstract] OR "sensory

Source	Concept	String as run
		deficit"[Title/Abstract] OR "sensory deficits"[Title/Abstract] OR "neurosensory deficit"[Title/Abstract] OR "neurosensory deficits"[Title/Abstract] OR "neurosensory disturbance"[Title/Abstract] OR "neurosensory disturbances"[Title/Abstract] OR "neurological complication"[Title/Abstract] OR "neurological complications"[Title/Abstract] OR "nerve complication"[Title/Abstract] OR "nerve complications"[Title/Abstract])
	Combination	Concept 1 AND Concept 2 AND Concept 3. English-language restriction applied at eligibility assessment.
Embase (Embase.com)	Concept 1	('third molar'/exp OR 'third molar':ti,ab,kw OR 'third molars':ti,ab,kw OR 'mandibular third molar':ti,ab,kw OR 'mandibular third molars':ti,ab,kw OR 'lower third molar':ti,ab,kw OR 'lower third molars':ti,ab,kw OR '3rd molar':ti,ab,kw OR '3rd molars':ti,ab,kw OR 'wisdom tooth':ti,ab,kw OR 'wisdom teeth':ti,ab,kw OR 'wisdom tooth extraction':ti,ab,kw OR 'third molar extraction':ti,ab,kw OR 'third molar surgery':ti,ab,kw OR m3m:ti,ab,kw OR mm3:ti,ab,kw OR 'tooth 38':ti,ab,kw OR 'tooth 48':ti,ab,kw)
	Concept 2	('panoramic radiography'/exp OR panoramic:ti,ab,kw OR 'panoramic radiograph':ti,ab,kw OR 'panoramic radiographs':ti,ab,kw OR 'panoramic radiography':ti,ab,kw OR 'panoramic imaging':ti,ab,kw OR orthopantomogram*:ti,ab,kw OR orthopantomograph*:ti,ab,kw OR opg:ti,ab,kw OR 'preoperative radiograph':ti,ab,kw OR 'preoperative radiographs':ti,ab,kw OR 'presurgical radiograph':ti,ab,kw OR 'presurgical radiographs':ti,ab,kw OR 'preoperative imaging':ti,ab,kw OR 'presurgical imaging':ti,ab,kw OR 'radiographic sign':ti,ab,kw OR 'radiographic signs':ti,ab,kw OR 'radiographic indicator':ti,ab,kw OR 'radiographic indicators':ti,ab,kw OR 'radiographic risk sign':ti,ab,kw OR 'radiographic risk signs':ti,ab,kw OR 'canal diversion':ti,ab,kw OR 'diversion of the canal':ti,ab,kw OR 'interruption of the white line':ti,ab,kw OR 'interruption of white line':ti,ab,kw OR 'darkening of the root':ti,ab,kw OR 'darkening of root':ti,ab,kw OR 'root darkening':ti,ab,kw OR 'root deflection':ti,ab,kw OR 'deflection of the root':ti,ab,kw OR 'root narrowing':ti,ab,kw OR 'narrowing of the root':ti,ab,kw OR 'canal narrowing':ti,ab,kw OR 'narrowing of the canal':ti,ab,kw OR 'dark and bifid apex':ti,ab,kw OR 'bifid root apex':ti,ab,kw)
	Concept 3	('inferior alveolar nerve'/exp OR 'inferior alveolar nerve':ti,ab,kw OR 'inferior alveolar nerve injury':ti,ab,kw OR 'inferior alveolar nerve injuries':ti,ab,kw OR 'inferior alveolar nerve damage':ti,ab,kw OR 'inferior alveolar nerve deficit':ti,ab,kw OR 'inferior alveolar nerve dysfunction':ti,ab,kw OR 'ian injury':ti,ab,kw OR 'ian damage':ti,ab,kw OR 'nerve injury':ti,ab,kw OR 'nerve damage':ti,ab,kw OR paresthesia:ti,ab,kw OR paraesthesia:ti,ab,kw OR hypoesthesia:ti,ab,kw OR hypoaesthesia:ti,ab,kw OR dysesthesia:ti,ab,kw OR dysaesthesia:ti,ab,kw OR 'sensory disturbance':ti,ab,kw OR 'sensory disturbances':ti,ab,kw OR 'sensory deficit':ti,ab,kw OR 'sensory deficits':ti,ab,kw OR 'neurosensory deficit':ti,ab,kw OR 'neurosensory deficits':ti,ab,kw OR 'neurosensory disturbance':ti,ab,kw OR 'neurosensory disturbances':ti,ab,kw)

Source	Concept	String as run
	Combination	#1 AND #2 AND #3
Scopus	Full string	TITLE-ABS-KEY("third molar" OR "third molars" OR "mandibular third molar" OR "mandibular third molars" OR "lower third molar" OR "lower third molars" OR "3rd molar" OR "3rd molars" OR "wisdom tooth" OR "wisdom teeth" OR "wisdom tooth extraction" OR "third molar extraction" OR "third molar surgery" OR M3M OR MM3 OR "tooth 38" OR "tooth 48") AND TITLE-ABS-KEY(panoramic OR orthopantomogram* OR orthopantomograph* OR OPG OR "panoramic radiograph" OR "panoramic radiographs" OR "panoramic radiography" OR "panoramic imaging" OR "preoperative radiograph" OR "preoperative radiographs" OR "presurgical radiograph" OR "presurgical radiographs" OR "preoperative imaging" OR "presurgical imaging" OR "radiographic sign" OR "radiographic signs" OR "radiographic indicator" OR "radiographic indicators" OR "radiographic risk sign" OR "radiographic risk signs" OR "canal diversion" OR "diversion of the canal" OR "interruption of the white line" OR "interruption of white line" OR "darkening of the root" OR "darkening of root" OR "root darkening" OR "root deflection" OR "deflection of the root" OR "root narrowing" OR "narrowing of the root" OR "canal narrowing" OR "narrowing of the canal" OR "dark and bifid apex" OR "bifid root apex") AND TITLE-ABS-KEY("inferior alveolar nerve" OR "inferior alveolar nerve injury" OR "inferior alveolar nerve injuries" OR "inferior alveolar nerve damage" OR "inferior alveolar nerve deficit" OR "inferior alveolar nerve dysfunction" OR "IAN injury" OR "IAN damage" OR "nerve injury" OR "nerve damage" OR paresthesia OR paraesthesia OR hypoesthesia OR hypoaesthesia OR dysesthesia OR dysaesthesia OR "sensory disturbance" OR "sensory disturbances" OR "sensory deficit" OR "sensory deficits" OR "neurosensory deficit" OR "neurosensory deficits" OR "neurosensory disturbance" OR "neurosensory disturbances")
Cochrane Library (CENTRAL)	Concept 1	("third molar" OR "third molars" OR "mandibular third molar" OR "mandibular third molars" OR "lower third molar" OR "lower third molars" OR "3rd molar" OR "3rd molars" OR "wisdom tooth" OR "wisdom teeth" OR (wisdom NEXT tooth NEXT extract*) OR (third NEXT molar NEXT extract*) OR (third NEXT molar NEXT surg*) OR M3M OR MM3 OR "tooth 38" OR "tooth 48")
	Concept 2	(panoramic OR orthopantomogram* OR orthopantomograph* OR OPG OR (panoramic NEXT radiograph*) OR (panoramic NEXT imaging) OR (preoperative NEXT radiograph*) OR (presurgical NEXT radiograph*) OR (pre-surgical NEXT radiograph*) OR (preoperative NEXT imaging) OR (presurgical NEXT imaging) OR (radiographic NEXT sign*) OR (radiographic NEXT indicator*) OR (radiographic NEXT risk NEXT sign*) OR "canal diversion" OR "diversion of the canal" OR "interruption of the white line" OR "interruption of white line" OR "darkening of the root" OR "darkening of root" OR "root darkening" OR "root deflection" OR "deflection of the root" OR "root narrowing" OR "narrowing of the root" OR "canal narrowing" OR "narrowing of the canal" OR "dark and bifid apex" OR "bifid root apex")
	Concept 3	("inferior alveolar nerve" OR (inferior NEXT alveolar NEXT nerve NEXT injur*) OR (inferior

Source	Concept	String as run
		NEXT alveolar NEXT nerve NEXT damag*) OR (inferior NEXT alveolar NEXT nerve NEXT deficit*) OR (inferior NEXT alveolar NEXT nerve NEXT dysfunction*) OR (IAN NEXT injur*) OR (IAN NEXT damag*) OR (nerve NEXT injur*) OR (nerve NEXT damag*) OR paresthesia OR paraesthesia OR hypoesthesia OR hypoaesthesia OR dysesthesia OR dysaesthesia OR (sensory NEXT disturbance*) OR (sensory NEXT deficit*) OR (neurosensory NEXT deficit*) OR (neurosensory NEXT disturbance*))
	Combination	#1 AND #2 AND #3. Truncation is nested with NEXT rather than placed inside quotation marks, which the CENTRAL interface rejects.
Google Scholar (supplementary)	Search approach	Simplified combinations of the three concepts — third molar + panoramic radiography + inferior alveolar nerve injury — run as successive queries, with results exported in batches of 20 (files GS 1.csv, GS 1–20, GS 21–40, GS 41–60, GS 61–80, GS 81–100, GS 101–111). Google Scholar does not support controlled vocabulary or nested Boolean syntax of the kind used above.
Reference-list and citation searching	Search approach	Reference lists of all included studies and of relevant previous systematic reviews were screened by hand; candidate records were exported via Zotero (file ZOTERO 1–20.csv).
Supplementary search — clinical utility	Additional concept combined with Concepts 1 and 2	(CBCT OR "cone beam computed tomography" OR "cone-beam computed tomography" OR "computed tomography" OR "3D imaging" OR "three-dimensional imaging" OR "clinical decision making" OR "clinical decision-making" OR "decision making" OR "surgical planning" OR "treatment planning" OR "preoperative planning" OR "clinical management" OR coronectomy OR referral). Run to address the secondary question on whether panoramic findings alter management. The comparator concept was not made mandatory in the primary search.

Table S5. Data audit, including the handling of the two internally inconsistent extracted rows and the un-stratified analysis

Part A reproduces every two-by-two table used in the analysis, with the unit of analysis and target condition for each study. Part B records the three faults identified by the internal consistency checks and how each was handled. Part C records the study-count audit. The analysis code that produced Tables 4 to 7 and Figures 2 to 4 accompanies this file.

Part A. Extracted two-by-two data

Study (year) [ref]	Country	Unit of analysis	Target condition	Radiographic sign	TP	TN	FP	FN
Huang 2015 [56]	China	Patient	Postoperative IAN injury	Darkening of the root	7	72	35	6
				Interruption of the white line	12	49	58	1
				Diversion of the canal	10	90	17	3
				Dark and bifid root apex	3	85	22	10
				Deflection of the root	4	83	24	9
				Narrowing of the canal	9	70	37	4
				Narrowing of the root	3	84	23	10
Gomes 2008 [53]	Brazil	Molar	Postoperative IAN injury	Darkening of the root	3	201	50	6
				Interruption of the white line	2	236	15	7
				Diversion of the canal	1	247	4	8
Rood 1990 [21]	UK	Molar	Postoperative IAN injury	Darkening of the root	8	701	38	13
				Interruption of the white line	5	709	30	16
				Diversion of the canal	6	727	12	15
				Narrowing of the root	0	737	2	21
Leung 2011 [34]	China	Tooth	Postoperative IAN injury	Darkening of the root	6	125	44	3
				Narrowing of the root	1	165	4	8
				Interruption of the white line	9	158	11	0

Study (year) [ref]	Country	Unit of analysis	Target condition	Radiographic sign	TP	TN	FP	FN
				Diversion of the canal	1	147	22	8
				Narrowing of the canal	0	160	9	9
Ghaeminia 2015 [16]	Netherlands	Molar	Postoperative IAN injury	Darkening of the root	15	214	74	5
				Interruption of the white line	5	215	73	15
Gallesio 2010 [54]	Italy	Patient	Postoperative IAN injury	Diversion of the canal	0	122	8	4
				Narrowing of the canal	0	124	6	4
				Deflection of the root	0	121	9	4
				Narrowing of the root	0	124	6	4
				Dark and bifid root apex	0	121	9	4
				Interruption of the white line	1	91	39	3
				Darkening of the root	3	98	32	1
Sedaghatfar 2005 [44]	USA	Molar	Intraoperative IAN exposure	Interruption of the white line	18	265	134	6
				Diversion of the canal	10	356	43	14
				Deflection of the root	4	344	55	20
				Narrowing of the root	12	361	38	12
Hasegawa 2013 [52]	Japan	Molar	Postoperative IAN injury	Interruption of the white line	9	399	13	19
				Diversion of the canal	11	395	17	17
Harada 2015 [30]	Japan	Patient	Postoperative IAN injury	Darkening of the root	3	174	91	12
				Interruption of the white line	3	124	141	12
				Diversion of the canal	0	259	6	15
				Dark and bifid root apex	0	262	3	15
				Deflection of the root	1	263	2	14

Study (year) [ref]	Country	Unit of analysis	Target condition	Radiographic sign	TP	TN	FP	FN
				Narrowing of the canal	0	255	10	15
				Narrowing of the root	0	263	2	15
Szalma 2010 [10] †	Hungary	Patient	Postoperative IAN injury	Darkening of the root	32	178	84	15

† Szalma et al. (2010) is excluded from the primary pooled analysis; see Part B.

Part B. Data corrections

#	Study	Sign	As extracted	Action taken	Basis and consequence
1	Ghaeminia et al. (2015) [16]	Narrowing of the canal	TP 0, TN 255, FP 10, FN 15	Row removed	Numerically identical to the Harada et al. (2015) row and carrying Harada's denominator (280) rather than Ghaeminia's (308). Judged a transcription duplicate; removing it prevents double counting. Narrowing of the canal is pooled from four studies rather than five.
2	Leung & Cheung (2011) [34]	Narrowing of the canal	TP 0, TN 169, FP 9, FN 9	TN corrected 169 → 160	Row summed to 187 against a study denominator of 178. The diseased count (9) is consistent with the study's other rows, so the error lies in the disease-negative cells. Consequential, and the four arithmetically admissible reconstructions do not agree: TN 169→160 gives DOR 2.67 (95% CI 0.98–7.28); FN 9→0 or FP 9→0 each give 3.48 (1.24–9.77); omitting the row gives 3.10 (1.06–9.01). The most conservative of the four is the one reported in Table 4 and in the abstract, and the full set is in Table S10. The discrepant cell cannot be resolved without the source data, so narrowing of the canal is treated throughout as a sign of unproven value.
3	Szalma et al. (2010) [10]	Darkening of the root	TP 32, TN 178, FP 84, FN 15	Retained in the primary analysis; examined in a leave-out sensitivity analysis	The published report describes a case–control sample of 41 cases and 359 controls (n = 400) with specificities of 85.5–96.9%; the extracted row implies n = 309, 47 events and a specificity of 67.9%. Only the sensitivity agrees closely (68.1% vs 68.3%). The study is retained in the primary analysis, and because a case–control design is rated high risk for patient selection under QUADAS-2 and sets its own sampling fraction, its influence is reported separately:

#	Study	Sign	As extracted	Action taken	Basis and consequence
					removing it changes only darkening of the root, from DOR 3.92 (95% CI 1.97–7.80) to 3.77 (1.57–9.08), and alters no conclusion. The extracted cells REQUIRE VERIFICATION against the source article.

Part C. Study-count audit

Count	Where it appeared	Status	Basis
33	Flow diagram and one sentence of the Results in the withdrawn submission	Superseded	Transcription error.
36	Table 1 of the withdrawn submission	Superseded	Szalma et al. (2010) was omitted from the table although it contributed two-by-two data to the meta-analysis; it has been added.
37	Manuscript, figures, tables and supplementary materials	Current	Verified against the Rayyan screening record. The full-text exclusion count is 70, not 71.

Table S6. Extended characteristics of all 37 included studies

Design, population, reference standard and target condition for each study are given in Table 1 of the manuscript. This table carries the remaining descriptive fields.

#	Study (year) [ref]	Intervention	Comparator / reference standard	Outcomes measured	Key findings	Limitations
1	Tantanapornkul et al. (2007) [27]	Cone-beam CT vs panoramic radiography	Intraoperative findings of neurovascular bundle exposure	Sensitivity, specificity, accuracy for predicting nerve exposure	CBCT sensitivity 93% and specificity 77% vs panoramic 70% and 63%	Cases excluded for severe bleeding; small sample; reliance on intraoperative findings
2	Khemla & Wongchuensoontorn (2022) [28]	Panoramic radiograph	CBCT (reference standard)	Sensitivity, specificity, PPV, NPV for IAN canal involvement	Panoramic sensitivity 83.1%, specificity 60%; darkening of root and interruption of white line linked to nerve involvement	Small subgroups (Class 3 ramus, distoangular); comparison limited by differing methodology
3	Elkhateeb & Awad (2018) [12]	Panoramic radiograph	CBCT (reference standard)	Sensitivity and specificity of panoramic signs for IAN canal proximity	Interruption of IAC wall, darkening of roots and narrowing of canal significantly correlated with CBCT contact	Female patients only; retrospective
4	Lee et al. (2015) [29]	3D-CT imaging	Panoramic radiography	Incidence of IAN damage, distance from IAC, correlation with proximity	IAN damage 0.35% with 3D-CT vs 1.1% without	Retrospective; selection bias; single centre
5	Guerrero et al. (2014) [17]	Panoramic radiography vs CBCT	Panoramic radiography	IAN sensory disturbance, swelling, trismus, haemorrhage, infection, alveolar osteitis	No significant difference in IAN injury between panoramic and CBCT; CBCT more accurate for root morphology	Single centre; sample size; no reduction in postoperative complications
6	Harada et al. (2015) [30]	Cone-beam CT	Panoramic radiography	Postoperative paresthesia; panoramic signs	Paresthesia most frequent with darkening of root and interruption of the canal; CBCT showed	Retrospective; no randomisation; possible observer bias; single 1-week

#	Study (year) [ref]	Intervention	Comparator / reference standard	Outcomes measured	Key findings	Limitations
					inter-radicular canal position as a significant factor	assessment
7	Nakamori et al. (2008) [31]	Panoramic radiography vs CT	Computed tomography	Distance between third molar and IAC, absence of cortication, IAN injury	Darkening of root and diversion of canal correlated with absence of cortication on CT	Retrospective; observer variability; single centre
8	Ghaeminia et al. (2015) [16]	Cone-beam CT	Panoramic radiography	IAN injury (temporary and permanent), complications, quality of life (OHIP-14), pain (VAS), emergency visits	No significant difference in IAN injury between CBCT and panoramic; CBCT did not reduce morbidity but improved surgical planning	Variability in surgeon experience; no reduction in IAN injury despite CBCT
9	Jhamb et al. (2009) [32]	Spiral computed tomography	Orthopantomography	Sensitivity, specificity, clinical findings, anatomical relationship	SCT outperformed OPG for detecting IAN proximity, but not significantly (P = 0.9871)	Small sample; possible observer bias; difference not statistically significant
10	Luo et al. (2018) [33]	Computed tomography	Panoramic radiography	Sensitivity, specificity, third molar–IAN relationship, injury risk	CT quantified the IAN relationship more accurately; OPG better detected close tooth–canal relationships	Small sample; no expert image evaluation; single centre
11	Leung & Cheung (2011) [34]	Panoramic radiography	Matched controls	IDN exposure, IDN deficit, predictive radiographic signs	Darkening of root strongly predictive of exposure and deficit	Sensitivity and specificity not computed for all signs
12	Hasani et al. (2017) [35]	Cone-beam CT	Panoramic radiography	Sensitivity, specificity, PPV, NPV for predicting IAN exposure	CBCT sensitivity 97.4% and PPV 92.7% vs panoramic 67.8% and 97.6%	Small sample; no control group for non-exposure cases; limited generalisability
13	Vranckx et al.	Panoramic	Panoramic	Postoperative	Anatomical variations of	Small sample; no

#	Study (year) [ref]	Intervention	Comparator / reference standard	Outcomes measured	Key findings	Limitations
	(2022) [36]	radiography vs CBCT	radiography	neurosensory disturbance, anatomical variations, root–nerve relation	the mandibular canal did not significantly predict IAN injury; CBCT superior for detecting variations	control group; self-reported complications
14	Korkmaz et al. (2017) [37]	Cone-beam CT	Panoramic radiography	Incidence of IAN injury, surgical outcomes, surgical time	CBCT reduced temporary IAN injury (4.2% vs 16.4%) but not long-term injury	Limited sample; no control for surgeon experience; high-risk cases only
15	Savani et al. (2018) [38]	Cone-beam CT	Panoramic radiography	Postoperative IAN injury, corticalization, anatomical relationship	CBCT more sensitive for detecting IAN injury risk with darkening of roots and loss of white line	Small sample; no control group; no long-term follow-up
16	Özden Yüce et al. (2021) [39]	Cone-beam CT	Panoramic radiography	Third molar–canal relationship, treatment decision, risk assessment	CBCT changed the treatment decision in 85 of 150 patients, often from extraction to coronectomy	Small sample; high-risk patients only; no long-term follow-up
17	Kubota et al. (2023) [40]	CBCT- and panoramic-featured prediction models	Panoramic-based prediction models	IAN injury risk, model performance, recalibration, discrimination, calibration	Extended CBCT model outperformed panoramic models; recalibrated C-statistic 0.822	Retrospective; selection bias; web-based calculator not used
18	Selvi et al. (2013) [41]	Preoperative CT (medical CT and CBCT)	Panoramic radiography	Postoperative IAN injury; personal, anatomical and radiographic risk factors	Increased age, female sex and larger cortical perforation were significant risk factors	Retrospective; selection bias; no standardisation of surgical experience
19	Jerjes et al. (2006) [42]	Panoramic radiography	Absence of radiographic signs	IAN injury (temporary and permanent), surgical difficulty prediction	Injury risk correlated with root darkening, deflection and interruption of the IAC; Pederson index effective	Retrospective; surgeon variability; single centre

#	Study (year) [ref]	Intervention	Comparator / reference standard	Outcomes measured	Key findings	Limitations
20	Winstanley et al. (2018) [13]	Panoramic radiography vs CBCT	Cone-beam CT	Tooth–canal contact; risk indicators on panoramic radiographs	Interruption of the white line and darkening of roots significantly associated with contact on CBCT	Small sample; selection bias; high-risk cases only
21	Guerrero et al. (2012) [43]	Cone-beam CT vs panoramic radiography	Panoramic radiography	Postoperative sensory disturbance; prediction of IAN exposure	No significant difference in sensory disturbance; CBCT superior for predicting exposure	Small sample; pilot design; no control group
22	Petersen et al. (2016) [18]	CBCT vs panoramic radiography	Panoramic radiography	Neurosensory disturbance (temporary and permanent)	No significant difference between CBCT (18.4%) and panoramic (11.2%) groups; relative risk 1.64	Underpowered; resource limitations; possible bias in surgeon behaviour
23	Sedaghatfar et al. (2005) [44]	Panoramic assessment of five signs	Absence of signs; clinician's preoperative risk assessment	Intraoperative IAN exposure; postoperative injury; sensitivity, specificity, PPV, NPV	IAN exposure in 5.7% of sites; four signs significantly associated ($P < .001$); PPV 0.12–0.24, NPV 0.96–0.99; risk rose with number of signs	Retrospective; single centre; outcome is exposure rather than injury; low event rate for permanent injury
24	Mansour et al. (2012) [45]	Panoramic radiography	IAN exposure during surgery	IAN proximity, radiographic signs, surgical and postoperative complications	Canal narrowing, root deviation and approximation predicted exposure and temporary injury	Retrospective; no control group; small sample; no standardised technique
25	Ghaeminia et al. (2009) [46]	CBCT vs panoramic radiography	CBCT as 3D reference; intraoperative exposure and postoperative injury	Sensitivity, specificity, accuracy for predicting IAN exposure	CBCT accurate for buccolingual canal position; similar sensitivity and specificity to panoramic, but better 3D visualisation	Small sample; high-risk cases; no significant accuracy difference; single centre
26	Susarla & Dodson (2007) [47]	Preoperative CT imaging	Panoramic radiography	IAN injury risk on panoramic and CT; intraoperative visualisation; postoperative	CT reclassified most cases from increased to low risk; no permanent nerve injury	Small sample; single centre; no control group; selection bias

#	Study (year) [ref]	Intervention	Comparator / reference standard	Outcomes measured	Key findings	Limitations
				sensory disturbance		
27	Bandiwadekar et al. (2024) [48]	Preoperative radiographs and intraoperative assessment	Intraoperative observation as reference	Root proximity to the IAC, injury risk, radiographic indicators	Root deflection, canal narrowing and radiographic disruption significantly associated with injury; higher risk in bony and mesioangular impactions	Retrospective; no control group; small sample; technique variability
28	Rood & Shehab (1990) [21]	Panoramic radiography	Postoperative sensory findings	Relationship of signs to nerve injury	Diversion of canal, darkening of root and interrupted white line were significant predictors	No pooled accuracy metrics; older imaging quality
29	Kapila et al. (2014) [49]	Panoramic radiography and CBCT	Comparison of panoramic and CBCT images	Sensitivity, specificity; juxta-apical radiolucencies vs canal position	CBCT clearer for root apices; no consistent relationship between juxta-apical radiolucencies and the canal	Small sample; retrospective; possible observer bias
30	Azahar et al. (2018) [50]	Panoramic radiography vs CBCT	Panoramic radiography	Accuracy for predicting IAN injury; interobserver reliability	CBCT more accurate for proximity, especially buccolingual position	Small sample; interobserver variability; single centre; no control group
31	Picoli et al. (2023) [51]	3D AI-driven model vs panoramic vs CBCT	CBCT and AI-based 3D models vs panoramic	Sensitivity, specificity, AUC; clinician confidence	AI models sensitivity 0.87, similar to CBCT 0.89 and above panoramic 0.73	Small sample; no significant difference between modalities; bilateral cases only; single centre
32	Hasegawa et al. (2013) [52]	Panoramic radiography vs computed tomography	CT classification; intraoperative and clinical outcomes	IAN injury; panoramic and CT findings; procedure-related factors	CT findings significantly associated with higher injury risk; loss of white line and canal diversion predictive on panoramic, but CT more accurate	Retrospective; no control group; selection bias; high radiation dose for CT

#	Study (year) [ref]	Intervention	Comparator / reference standard	Outcomes measured	Key findings	Limitations
33	Gomes et al. (2008) [53]	Panoramic radiography	Surgical outcomes	Sensitivity, specificity, PPV, NPV, nerve injury	Sensitivity 66%, specificity 39%, NPV 0.97; certain signs predictive	Limited specificity; operator-dependent interpretation
34	Gallesio et al. (2010) [54]	Panoramic radiography	Intraoperative findings	Nerve exposure, postoperative hypoesthesia	Bifid apex and radiolucent apex associated with higher risk	Diagnostic accuracy values not reported; small sample
35	Ruiz-Roca et al. (2024) [55]	Panoramic radiography (Rood & Shehab / Koong criteria)	CBCT as reference standard	Diagnostic validity indices; postoperative neurosensory change; odds ratios	Sensitivity 81.2%, specificity 33.2%, PPV 51.2%, NPV 67.1% for CBCT-confirmed contact; darkening plus interruption raised risk (OR 3.07); mean paraesthesia ~79 days; no permanent injuries	Retrospective; single centre; low specificity; selection bias toward high-risk cases; limited generalisability
36	Huang et al. (2015) [56]	Panoramic radiography	Clinical neurosensory examination	Sensitivity, specificity, PPV, NPV, postoperative neurosensory deficit	Darkening of root and narrowing of canal showed high sensitivity; moderate specificity	Retrospective; limited control of confounders
37	Szalma et al. (2010) [10]	Panoramic radiography (classic signs, root curvature, IAC–root overlap)	Controls without postoperative paraesthesia	IAN paraesthesia; sensitivity, specificity, PPV, NPV	Interruption of the superior cortex, diversion of the canal and darkening of the root significantly associated with paraesthesia ($P < .001$); sensitivities 14.6–68.3%, specificities 85.5–96.9%	Case-control design (41 cases, 359 controls); retrospective; prevalence not that of the source population

Table S7. Source and rationale for every QUADAS-2 domain judgement

Domain-level judgements are given in Table 8 of the manuscript. Full text could not be obtained for Rood and Shehab (1990), Leung and Cheung (2011), Sedaghatfar et al. (2005) or Szalma et al. (2010); those assessments rest on the published abstract and reported design, and domains not addressed in the available report are rated Unclear.

Study (year) [ref]	Source used for the assessment	Basis for the judgements
Huang 2015 [56]	submitted manuscript	Panoramic radiography only; low risk for selection, index test and flow/timing. Reference standard at high risk owing to absence of blinding of neurosensory assessment.
Gomes 2008 [53]	submitted manuscript	Enrolment not described. Outcome assessment unblinded; index-test interpretation blinding not reported.
Rood 1990 [21]	abstract + MeSH (PMID 2322523)	Combined retrospective and prospective surveys; enrolment and consecutiveness not described. Blinding of radiographic interpretation not reported. Method of postoperative sensory assessment not standardised or described. Interval between index test and reference standard not stated for the retrospective component.
Leung 2011 [34]	study design as extracted; full text not retrieved	Prospective cohort of consecutive third molar surgical patients; case-control design avoided. Blinding of radiographic interpretation to nerve status not reported. Method of neurosensory assessment not described in the material available.
Ghaeminia 2015 [16]	submitted manuscript	Randomised multicentre trial; appropriate enrolment and standardised imaging. Reference standard at high risk: nerve status self-reported by unblinded participants.
Gallesio 2010 [54]	submitted manuscript	Patients included on the basis of a positive panoramic finding, so enrolment is conditioned on the index test. Blinding of outcome assessment not reported.
Sedaghatfar 2005 [44]	abstract (PMID 15635549)	Retrospective cohort of 423 consecutive mandibular third molars evaluated and removed; case-control design avoided. Index-test blinding not reported, and in a retrospective record review the radiographic sign and the operative note are held in the same record. Reference standard is direct intraoperative visualisation recorded by the operating surgeon, who had seen the radiograph. Applicability of the reference standard is a high concern because the target condition is nerve EXPOSURE, not postoperative nerve injury.
Hasegawa 2013 [52]	full text (Int J Oral Maxillofac Surg 2013;42:843-51)	440 of 2528 teeth selected because they were close to the canal on panoramic radiography or were expected to be difficult to extract, so enrolment is conditioned on the index test and the sample is enriched for risk. Index test at low risk: panoramic and CT findings were read independently by two authors who were 'blinded independently to the postoperative IANI status'. Reference standard used pin-prick and light-touch testing with neurological examination and follow-up to six months, but blinding of the assessor to the panoramic findings is not reported.
Harada 2015 [30]	full text (Bull Tokyo Dent	Only patients whose roots contacted or were superimposed on the canal on panoramic

**Study (year) [ref]**	**Source used for the assessment**	**Basis for the judgements**
	Coll 2015;56:1-8)	radiography were carried forward, so enrolment is conditioned on the index test. Three observers read the images with consensus resolution, but blinding to nerve status is not stated. Paresthesia was 'reported' at one week postoperatively with no standardised neurosensory protocol described. 186 of 466 imaged patients did not proceed to extraction, and assessment was at a single one-week timepoint.
Szalma 2010 [10]	abstract (PMID 19846324)	CASE-CONTROL DESIGN: 41 cases with postoperative paresthesia and 359 controls without. QUADAS-2 rates a case-control design as high risk for patient selection by definition, and the sampling fraction is set by the investigators rather than by the clinical population, so prevalence and both predictive values derived from this study are not generalisable. Blinding of radiographic interpretation and the method of paresthesia ascertainment are not described in the available report.

Table S8. Operational reference-standard definitions for the ten studies contributing two × two data

Each field reproduces what the primary report states. Items the report does not state are recorded as "Not reported" rather than inferred. Full text could not be obtained for Rood and Shehab (1990), Leung and Cheung (2011), Sedaghatfar et al. (2005) or Szalma et al. (2010); those rows rest on the published abstract and the reported design.

Study (year) [ref]	Target condition	Reference standard as reported	Tests specified	Assessment interval	Readers of the index test	Index test blinded to outcome	Source
Huang et al. (2015) [56]	Postoperative IAN injury	Clinical neurosensory examination of the lower lip and chin	Not specified	Not reported	Not reported	Not reported	Full text
Gomes et al. (2008) [53]	Postoperative IAN injury	Clinical neurosensory examination	Not specified	Not reported	Not reported	Not reported	Full text
Rood & Shehab (1990) [21]	Postoperative IAN injury	Postoperative sensory assessment; neither standardised nor described	Not specified	Not reported	Not reported	Not reported	Abstract and indexing record (PMID 2322523)
Leung & Cheung (2011) [34]	Postoperative IAN injury	Postoperative neurosensory deficit; method not described in the available report	Not specified	Not reported	Not reported	Not reported	Design as extracted; full text not retrieved
Ghaeminia et al. (2015) [16]	Postoperative IAN injury	Altered sensation self-reported by trial participants, who were not blinded to the imaging received	Patient report	Not reported	Not reported	Not reported	Full text
Gallesio et al. (2010) [54]	Postoperative IAN injury	Clinical neurosensory examination	Not specified	Not reported	Not reported	Not reported	Full text
Sedaghatfar et al. (2005) [44]	Intraoperative IAN exposure	Direct visualisation of the neurovascular bundle in the socket, recorded by the operating surgeon	Not applicable	At operation	Not reported	No; the surgeon had seen the radiograph	Abstract (PMID 15635549)
Hasegawa et al. (2013) [52]	Postoperative IAN injury	Pin-prick and light-touch testing with neurological examination	Pin-prick; light touch	Follow-up to 6 months	Two authors, independently	Yes, for the index test; assessor blinding for the reference standard not	Full text

Study (year) [ref]	Target condition	Reference standard as reported	Tests specified	Assessment interval	Readers of the index test	Index test blinded to outcome	Source
						reported	
Harada et al. (2015) [30]	Postoperative IAN injury	Paraesthesia as reported by the patient; no standardised neurosensory protocol described	Not specified	1 week	Three observers, consensus resolution	Not reported	Full text
Szalma et al. (2010) [10]	Postoperative IAN injury	Postoperative paraesthesia; method of ascertainment not described	Not specified	Not reported	Not reported	Not reported	Abstract (PMID 19846324)

Table S9. Leave-one-out analyses with diagnostic odds ratio and likelihood-ratio ranges

Part A. Leave-one-out analysis for every sign

Each row omits one contributing study and re-pools the remainder. Both likelihood ratios are reported alongside the diagnostic odds ratio, because a confidence interval that excludes 1 does not by itself establish that a sign shifts probability enough to alter management. No sign reaches a negative likelihood ratio near 0.1 in any iteration.

Radiographic sign	Study omitted	k	DOR (95% CI)	LR+	LR−	CI excludes 1
Darkening of the root	Huang 2015	7	4.20 (1.93–9.12)	2.44	0.60	Yes
Darkening of the root	Gomes 2008	7	4.25 (2.00–9.02)	2.38	0.58	Yes
Darkening of the root	Rood 1990	7	3.25 (1.59–6.66)	2.03	0.60	Yes
Darkening of the root	Leung 2011	7	3.72 (1.72–8.06)	2.29	0.62	Yes
Darkening of the root	Ghaeminia 2015	7	3.43 (1.59–7.41)	2.19	0.65	Yes
Darkening of the root	Gallesio 2010	7	3.69 (1.77–7.66)	2.28	0.62	Yes
Darkening of the root	Harada 2015	7	5.32 (3.34–8.47)	2.71	0.53	Yes
Darkening of the root	Szalma 2010	7	3.77 (1.57–9.08)	2.29	0.64	Yes
Darkening of the root — range across iterations			3.25–5.32	2.03–2.71	0.53–0.65	Yes throughout
Interruption of the white line	Huang 2015	7	3.54 (0.86–14.52)	2.28	0.82	No
Interruption of the white line	Gomes 2008	7	3.95 (0.92–17.03)	2.23	0.73	No
Interruption of the white line	Rood 1990	7	3.67 (0.80–16.91)	2.17	0.73	No
Interruption of the white line	Leung 2011	7	2.65 (0.76–9.25)	1.78	0.84	No
Interruption of the white line	Ghaeminia 2015	7	5.05 (1.18–21.63)	2.80	0.70	Yes
Interruption of the white line	Gallesio 2010	7	4.83 (1.20–19.37)	2.72	0.72	Yes
Interruption of the white line	Hasegawa 2013	7	3.17 (0.79–12.77)	2.07	0.75	No
Interruption of the white line	Harada 2015	7	6.01 (1.93–18.77)	3.26	0.68	Yes
Interruption of the white line — range across iterations			2.65–6.01	1.78–3.26	0.68–0.84	No — lost when omitting: Huang 2015, Gomes 2008, Rood 1990, Leung 2011, Hasegawa 2013
Diversion of the canal	Huang 2015	6	6.34 (2.10–19.18)	5.86	0.81	Yes
Diversion of the canal	Gomes 2008	6	7.82 (2.82–21.75)	5.37	0.75	Yes

Radiographic sign	Study omitted	k	DOR (95% CI)	LR+	LR−	CI excludes 1
Diversion of the canal	Rood 1990	6	5.86 (2.01–17.09)	4.20	0.81	Yes
Diversion of the canal	Leung 2011	6	13.33 (6.83–26.01)	7.56	0.73	Yes
Diversion of the canal	Gallesio 2010	6	9.36 (3.73–23.50)	6.41	0.75	Yes
Diversion of the canal	Hasegawa 2013	6	5.99 (1.78–20.12)	4.49	0.82	Yes
Diversion of the canal	Harada 2015	6	9.72 (3.96–23.87)	6.09	0.72	Yes
Diversion of the canal — range across iterations			5.86–13.33	4.20–7.56	0.72–0.82	Yes throughout
Narrowing of the canal	Huang 2015	3	1.12 (0.21–6.12)	1.13	0.99	No
Narrowing of the canal	Leung 2011	3	3.10 (1.06–9.01)	2.11	0.88	Yes
Narrowing of the canal	Gallesio 2010	3	2.75 (0.95–7.96)	1.64	0.93	No
Narrowing of the canal	Harada 2015	3	3.16 (1.08–9.22)	2.19	0.86	Yes
Narrowing of the canal — range across iterations			1.12–3.16	1.13–2.19	0.86–0.99	No — lost when omitting: Huang 2015, Gallesio 2010
Narrowing of the root	Huang 2015	4	4.15 (1.02–16.86)	4.04	0.95	Yes
Narrowing of the root	Rood 1990	4	1.84 (0.65–5.17)	3.37	0.89	No
Narrowing of the root	Leung 2011	4	1.72 (0.58–5.09)	3.70	0.93	No
Narrowing of the root	Gallesio 2010	4	2.10 (0.75–5.91)	4.78	0.91	No
Narrowing of the root	Harada 2015	4	1.99 (0.71–5.60)	4.18	0.89	No
Narrowing of the root — range across iterations			1.72–4.15	3.37–4.78	0.89–0.95	No — lost when omitting: Rood 1990, Leung 2011, Gallesio 2010, Harada 2015
Deflection of the root	Huang 2015	2	4.39 (0.66–29.38)	2.95	0.95	No
Deflection of the root	Gallesio 2010	2	2.76 (0.52–14.49)	3.74	0.86	No
Deflection of the root	Harada 2015	2	1.52 (0.47–4.86)	2.01	0.84	No
Deflection of the root — range across iterations			1.52–4.39	2.01–3.74	0.84–0.95	No — lost when omitting: Huang 2015, Gallesio 2010, Harada 2015
Dark and bifid root apex	Huang 2015	2	1.85 (0.22–15.47)	1.68	0.98	No
Dark and bifid root apex	Gallesio 2010	2	1.32 (0.38–4.59)	2.16	0.93	No
Dark and bifid root apex	Harada 2015	2	1.20 (0.34–4.18)	1.60	0.91	No

Radiographic sign	Study omitted	k	DOR (95% CI)	LR+	LR−	CI excludes 1
Dark and bifid root apex — range across iterations			1.20–1.85	1.60–2.16	0.91–0.98	No — lost when omitting: Huang 2015, Gallesio 2010, Harada 2015

Part B. Sensitivity analysis restricted to patient-level studies

Restricted to Huang et al. (2015), Gallesio et al. (2010), Harada et al. (2015) and Szalma et al. (2010), the four studies that used the patient as the unit of analysis. Removing the five tooth-level studies removes about two thirds of the data.

Radiographic sign	k	N	Sensitivity	Specificity	DOR (95% CI)	CI excludes 1
Darkening of the root	4	843	0.53	0.68	2.40 (0.78–7.36)	No
Interruption of the white line	3	534	0.49	0.54	1.10 (0.11–11.37)	No
Diversion of the canal	3	534	0.22	0.93	4.95 (0.75–32.59)	No
Narrowing of the canal	3	534	0.20	0.90	3.10 (1.06–9.01)	Yes
Narrowing of the root	3	534	0.15	0.95	1.42 (0.45–4.50)	No
Deflection of the root	3	534	0.18	0.94	2.12 (0.74–6.07)	No
Dark and bifid root apex	3	534	0.15	0.94	1.33 (0.42–4.21)	No

Part C. Sensitivity analysis excluding Szalma et al. (2010)

Values are shown as primary analysis → analysis with Szalma et al. (2010) removed. Szalma et al. contribute data for darkening of the root only, so no other estimate changes.

Radiographic sign	k	Sensitivity	Specificity	DOR (95% CI)	Effect on conclusion
Darkening of the root	8 → 7	0.54 → 0.50	0.77 → 0.78	3.92 (1.97–7.80) → 3.77 (1.57–9.08)	No change
Interruption of the white line	8 → 8	0.37 → 0.37	0.84 → 0.84	3.96 (1.09–14.39) → 3.96 (1.09–14.39)	No change
Diversion of the canal	7 → 7	0.27 → 0.27	0.95 → 0.95	8.18 (3.31–20.20) → 8.18 (3.31–20.20)	No change
Narrowing of the canal	4 → 4	0.15 → 0.15	0.92 → 0.92	2.67 (0.98–7.28) → 2.67 (0.98–7.28)	No change
Narrowing of the root	5 → 5	0.12 → 0.12	0.97 → 0.97	2.10 (0.79–5.60) → 2.10 (0.79–5.60)	No change
Deflection of the root	3 → 3	0.18 → 0.18	0.94 → 0.94	2.12 (0.74–6.07) → 2.12 (0.74–6.07)	No change
Dark and bifid root apex	3 → 3	0.15 → 0.15	0.94 → 0.94	1.33 (0.42–4.21) → 1.33 (0.42–4.21)	No change

Table S10. Arithmetically admissible reconstructions of the Leung and Cheung (2011) [23] narrow-ing-of-the-canal row

The four extracted cells sum to 187 against a reported denominator of 178. Four handlings are arithmetically admissible. Reconstruction A, the only one that does not yield a confidence interval excluding 1, is the one reported in Table 4 and in the abstract. The discrepancy cannot be resolved without the source data.

	Reconstruction	Cells used for Leung & Cheung	k	N	Pooled sensitivity	Pooled specificity	Pooled DOR (95% CI)	CI excludes 1
A	TN reduced 169 -> 160 (row sums to the published N = 178)	TP 0, TN 160, FP 9, FN 9	4	712	0.15	0.92	2.67 (0.98–7.28)	No
B	FN reduced 9 -> 0 (canal narrowing absent in every injured case)	TP 0, TN 169, FP 9, FN 0	4	712	0.26	0.92	3.48 (1.24–9.77)	Yes
C	FP reduced 9 -> 0 (no sign-positive uninjured case)	TP 0, TN 169, FP 0, FN 9	4	712	0.15	0.95	3.48 (1.24–9.77)	Yes
D	Leung row excluded entirely	row omitted	3	534	0.20	0.90	3.10 (1.06–9.01)	Yes
